# Supplementary material for: Sequence and Structure-Based Analysis of Specificity Determinants in Eukaryotic Protein Kinases
Source: Cell Rep. 2021 Jan 12;34(2):108602. doi: 10.1016/j.celrep.2020.108602 (PMC7809594; doi:10.1016/j.celrep.2020.108602)
Supplement: Document S1. Figures S1–S6 and Tables S2, S4, and S6 [file mmc1.pdf]

Cell Reports, Volume *34*

Supplemental Information

Sequence and Structure-Based Analysis of Specificity Determinants in Eukaryotic Protein Kinases

David Bradley, Cristina Viéitez, Vinothini Rajeeve, Joel Selkrig, Pedro R. Cutillas, and Pedro Beltrao

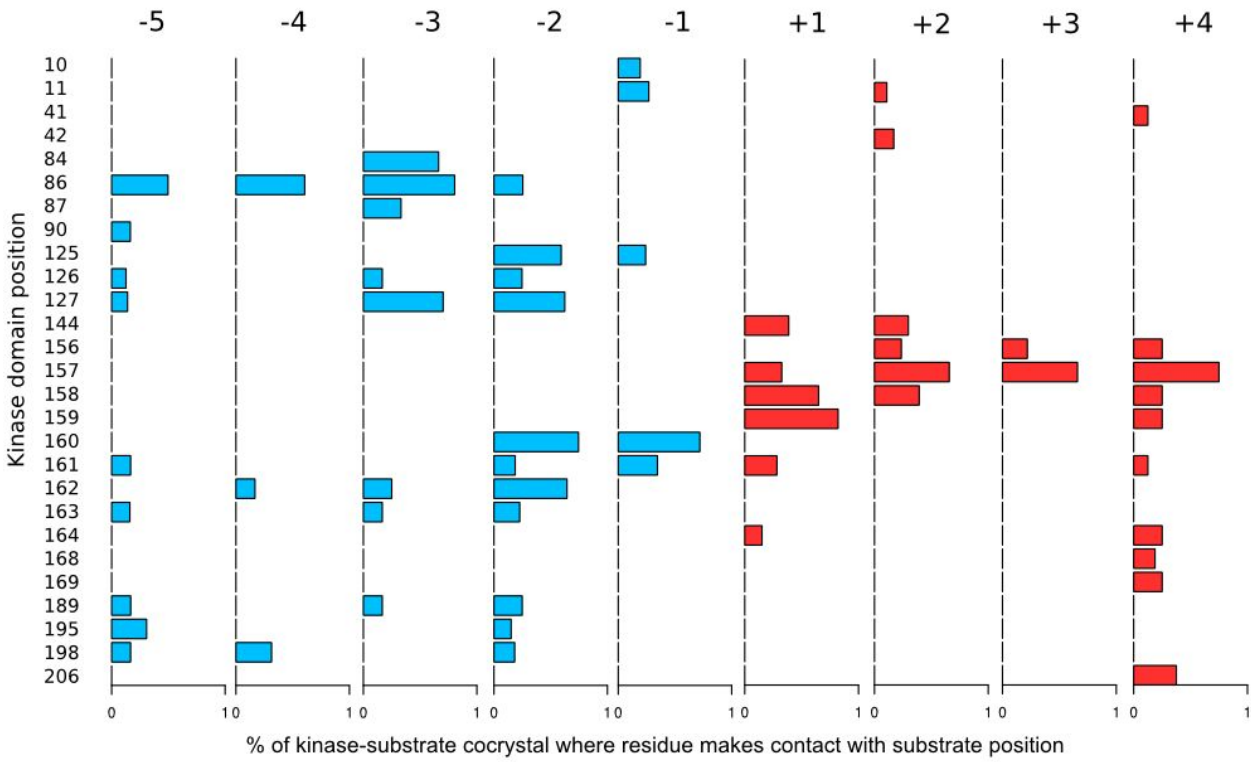

Figure S1. Substrate binding profile of Ser/Thr kinases, Related to Figure 1 - Binding prevalence for Ser/Thr kinase residues (mapped to the eukaryotic protein kinase domain, PFAM: PF00069), in terms of proportion of Ser/Thr kinase-substrate cocrystal structures in which the residue is found to contact (within 4Å) the substrate at a given position (e.g. +1 position). Sets of homologous kinases (e.g. AKT1 and AKT2) were counted once only.

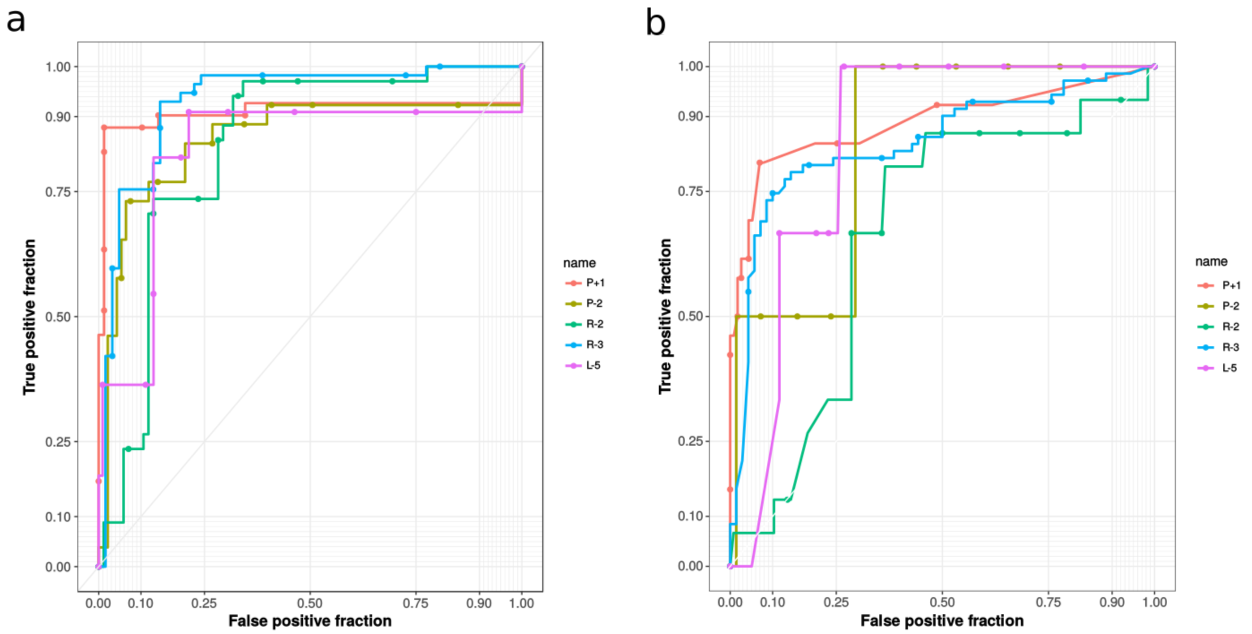

Figure S2. Benchmark performance of naive Bayes specificity predictors, Related to STAR Methods - Receiver Operating Characteristic (ROC) curves for five specificity classifiers. a) Naive Bayes classifiers for P+1, P-2, R-2, R-3, and L-5 preferences were assessed using leave-one-out cross-validation b) Naive Bayes classifiers for P+1, P-2, R-2, R-3, and L-5 preferences were assessed using an independent test set of 141 S/T PWMs from (Sugiyama, Imamura and Ishihama, 2019).

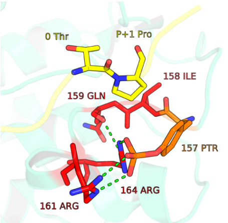

Proline at position +1: The +1 proline amide group is unable to act as an H-bond donor to the backbone carbonyl at 159. In absence of this interaction (which occurs in non-Pro+1 kinases with glycine at 159) the arginine side chain at 164 serves as an H-bond donor to the backbone carbonyl at 159. In many Pro+1 kinases the arginine side chain is stabilised by a negative side group (157 pTyr here), which in turn forms polar contacts (2.6Å) with arginine at 161 in this example. Isoleucine at 158 is unlikely to be a direct specificity determinant, but instead probably contributes to the CMGC-specific stabilisation of the kinase via non-bonded contacts with activation segment residues.

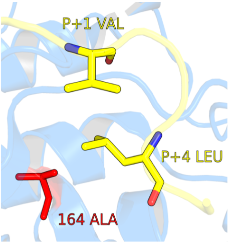

Leucine at position +4: Our analysis suggests that a substitution of isoleucine/leucine/methionine for alanine at position 164 contributes to selectivity for leucine at position +4. Mutation to alanine will result in a loss of packing interactions with the hydrophobic side chain at position +1. This is probably compensated for by a hydrophobic residue at position +4, which in the structure PDB: 3IEC can be observed to pack against the +1 valine in the +1 pocket (3.9Å).

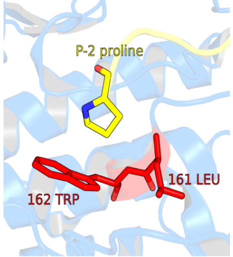

Proline at position -2: kinases with this preference usually feature a bulky residue at position 162 (tyrosine or tryptophan), and either leucine or arginine at position 161. Hydrophobic contacts (161: 3.4Å, 162: 3.7Å) between these side chains and the proline side group likely confers modest Pro-2 selectivity. Our analysis also suggests that this preference overlaps with modest Leu-2 selectivity.

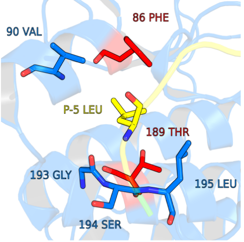

Leucine at position -5: The aromatic side chain at position 86 packs against the hydrophobic residue at substrate position -5 (3.4Å). The absence of a negatively-charged glutamate residue at 189 is another factor that favours the binding of a hydrophobic residue at position -5. The other positions highlighted -- 90, 193, 194, 195 -- also constitute the -5 binding pocket and may be important. In addition to the evidence given in **Figure 3**, recent experimental evidence also strongly suggests that position 189 is an SDR for the L-5 specificity (Chen *et al.*, 2017).

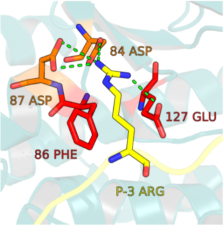

Arginine at position -3: position 86 mainly features a tyrosine or phenylalanine residue, which packs against the hydrophobic moiety of the arginine side chain. The glutamate at 127 can be observed to bond with R-3 in a few co-crystal structures in which R-2 or R-5 is not also present. Contact with aspartate/glutamate at 84 (2.8Å) is also observed in many co-crystal structures and has been validated as an SDR (Gibbs and Zoller, 1991; Huang *et al.*, 1995), but is not necessary for R-3 selectivity in all kinases. Polar contacts between R-3 and aspartate at position 87 (2.9Å) have so far been observed in CAMK kinases only (Pogacic *et al.*, 2007; Nesić *et al.*, 2010). In CMGC kinases, the mode of binding is similar to that of R-2 binding in AGC kinases, with glutamates at 127 and 189 (3.0Å and 2.6Å, respectively) forming polar contacts with the substrate arginine.

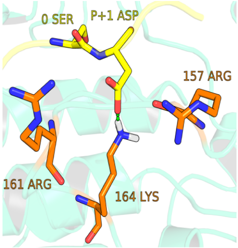

Aspartate/glutamate at position +1 (CMGC): The construction of a structural model of a CSNK2A1-peptide complex suggest that positively-charged arginine residues at positions 157 and 161, and a lysine residue at position at 164, are important for aspartate/glutamate selectivity. The suggested role of 164 lysine in particular is supported by a previous experimental study (Sarno *et al.*, 1997).

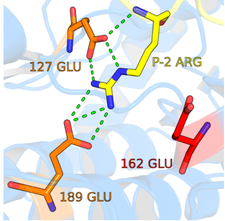

Arginine at position -2: Glutamate at positions 127 and 189 are together strongly associated with R-2 selectivity (Zhu *et al.*, 2005; Ben-Shimon and Niv, 2011). The alignment-based method presented here however does not account for positional inter-dependency. Position 162 usually features a glutamate in R-2 kinases, although this residue contacts R-6 directly (2.9Å) rather than R-2. A previous study however suggests a role for 162 in substrate recognition beyond its interaction with R-6 (Moore, Adams and Taylor, 2003).

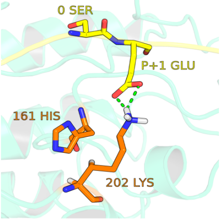

Aspartate/glutamate at position +1 (AGC): The construction of a structural model of a GRK2-peptide complex implicates the positively-charged lysine residue at position 202 as a determinant of +1 aspartate/glutamate selectivity. The lysine side chain at position 202 form polar contacts with the glutamate side chain at +1 in the constructed model.

Figure S3. Structural rationalisation of several kinases SDRs, related to Figure 2 and Figure 3 - Eight different kinase position preferences were rationalised using empirical or homology-based (D/E+1 CMGC and D/E+1 AGC) kinase-substrate models. Putative SDRs identified from kinase alignments (**Figure 1c** and **Figure 2**) are coloured in red and all other potential SDRs in orange. Substrate residues are coloured in yellow.

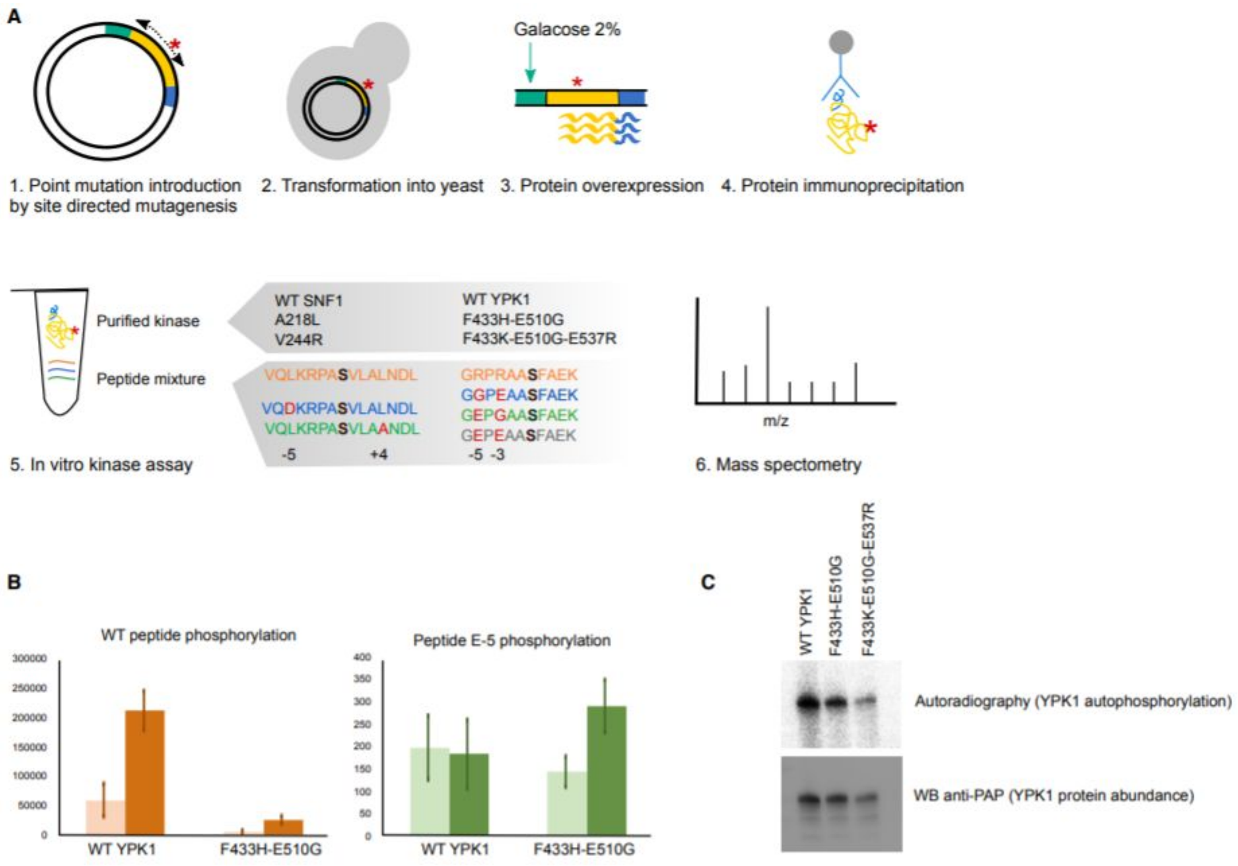

Figure S4. Experimental validation of kinase SDRs in yeast, Related to Figure 3 and Figure 6 - a) Experimental setup used to introduce point mutations in Snf1 and Ypk1-tagged kinases in plasmids, overexpression in yeast, protein purification and in *vitro kinase* assays. For the Ypk1 assay, every kinase (WT or mutant) was individually tested with a pool of peptides: WT (orange), mutated at R-3 (blue), mutated at R-5 (green) and mutated at R-3 and R-5 (grey). Incorporation of γ-32P ATP was measured by mass spectrometry. The residue numbers 433, 510, and 537 correspond to domain positions 86, 162, and 189, respectively. b) Phosphopeptide quantification after the Ypk1 kinase assay for 0 min (light color) and 30 min (dark color) at 30C (median and standard deviation for 3 biological replicates) c) Ypk1 kinase activity shown as kinase autophosphorylation after 30 min incubation with γ-32P ATP (autoradiography). Protein abundance shown by western blot using anti-PAP antibody.

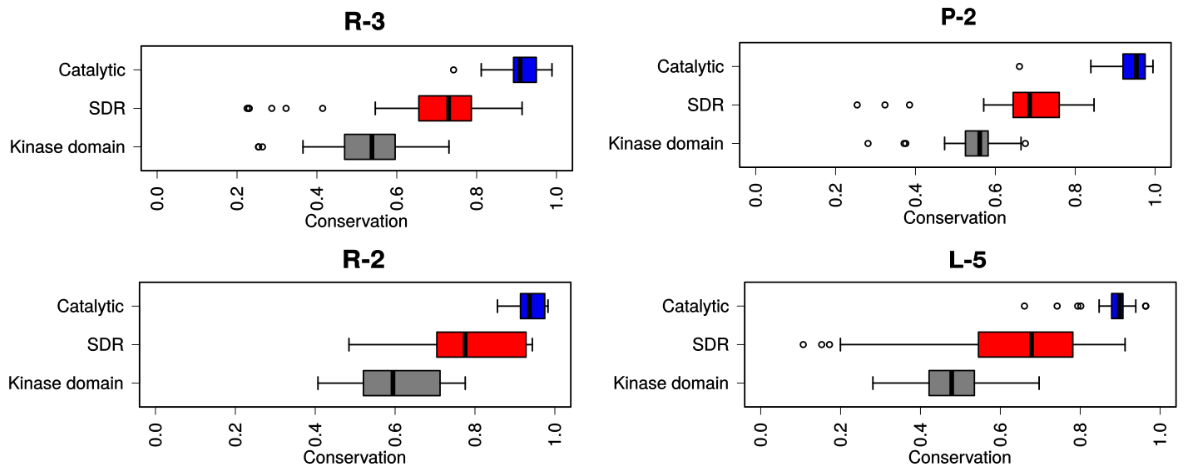

Figure S5. Conservation of kinase SDRs, catalytic residues, and domain residues between orthologs, Related to Figure 5 - Conservation of domain residues, SDRs, and catalytic residues for the R-3, P-2, R-2, and L-5 specificities. Each data point represents the average conservation (among kinase domain positions, SDR, or catalytic residues) for an alignment of orthologous kinases where the human kinase is a predicted R-3, P-2, R-2, or L-5 kinase.

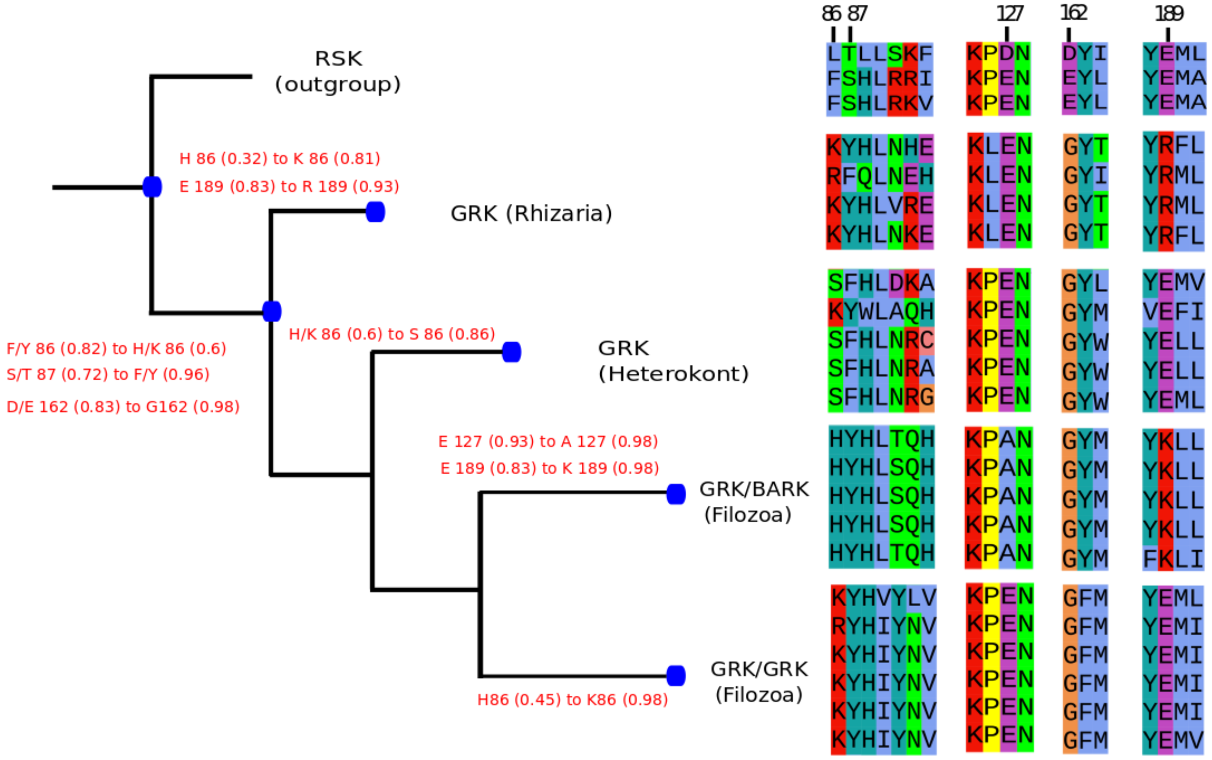

Figure S6. Ancestral sequence reconstruction for the GRK family, related to Figure 6 - Topology of the GRK phylogeny (left) with a sample of the GRK multiple sequence alignment (right). Labels at the selected nodes (blue circles) represent the reconstructed probabilities of amino acids from the antecedent blue node to the labelled node. The labelled domain positions are found in the -2/-3 binding pocket and are discussed in the main text (**Figure 6c**).

PDB	Kinase	Group	Kinase species	Substrate	Resolution (Å)	Publication	-5	-4	-3	-2	-1	0	1	2	3	4	5
2cpk*	PKA	AGC	Human	PKI-alpha	2.7	Knighton *et al.*, 1991	T	G	R	R	N	**A**	I	H	D	x	x
4o21*	PKA	AGC	*M. musculus*	PKI-alpha	1.95	Gerlits *et al.*, 2014	T	G	R	R	A	**S**	I	H	D	x	x
4wih	PKA	AGC	*C. griseus*	PKI-alpha	1.1	Kudlinzki *et al.*, 2015	T	G	R	R	Q	**A**	I	H	D	I	x
4xw5*	PKA	AGC	*M. musculus*	PKI-alpha	1.82	Gerlits *et al.*, 2015	T	G	R	R	A	**C**	I	H	D	x	x
3o7l	PKA	AGC	*M. musculus*	Pln	2.8	Masterson *et al.*, 2010	A	I	R	R	A	**S**	T	I	x	x	x
2phk	PHKg	CAMK	*O. cuniculus*	Synthetic	2.6	Lowe *et al.*, 1997	x	x	R	Q	M	**S**	F	R	L	x	x
1qmz	CDK2	CMGC	Human	Synthetic	2.2	Brown *et al.*, 1999	x	x	H	H	A	**S**	P	R	K	x	x
3qhr	CDK2	CMGC	Human	Synthetic	2.2	Bao *et al.*, 2011	x	x	x	P	K	**T**	P	K	K	A	K
1o6k	AKT-2	AGC	Human	GSK3-beta	1.7	Yang *et al.*, 2002	R	P	R	T	T	**S**	F	A	E	x	x
3cqw	AKT-1	AGC	Human	Synthetic	2.0	Lippa *et al.*, 2008	R	P	R	T	T	**S**	F	A	E	x	x
3mvh	v-AKT	AGC	Human	Synthetic	2.01	Freeman-Cook *et al.*, 2010	R	P	R	T	T	**S**	F	A	E	x	x
2c3i	PIM-1	CAMK	Human	Synthetic	1.9	Pogacic *et al.*, 2007	R	R	R	H	P	**S**	G	x	x	x	x
4dc2	PKC-i	AGC	*M. musculus*	Par-3	2.4	Wang *et al.*, 2012	G	F	G	R	Q	**S**	M	S	x	x	x
2wo6	DYRK-1A	CMGC	Human	Crb-2	2.5	Soundararajan *et al.*, 2013	x	A	R	P	G	**T**	P	A	L	x	x
4jdh	PAK-4	STE	Human	Synthetic	2	Chen *et al.*, 2014	x	x	R	R	R	**T**	W	Y	F	G	G
4l67*	PAK-4	STE	Human	Pak4	2.8	Wang *et al.*, 2013	A	R	R	P	K	**P**	L	V	D	P	A
4ouc	Haspin	Other	Human	Histone H3.2	1.9	Maiolica *et al.*, 2014	x	x	x	A	R	**T**	K	Q	T	A	x
3kl8*	CAMKII	CAMK	*C .elegans*	CAMK2n1	3.37	Chao *et al.*, 2010	I	G	R	S	K	**R**	V	V	I	x	x
3iec*	MARK2	CAMK	*H. sapiens*	cagA	2.2	Nesic *et al.*, 2010	L	K	R	H	D	**K**	V	D	D	L	S
3tl8	BAK1	N/A	*A. thaliana*	HoAB2	2.5	Cheng *et al.*, 2011	I	D	L	G	E	**S**	L	V	Q	H	P

Table S2 - List of PDB structures featuring Ser/Thr protein kinases in complex with a substrate peptide/protein at the active site, Related to STAR Methods. In *4dc2* (PKC-i), the substrate peptide N-terminal to the phosphoacceptor ‘loops out’ from the active site to form a non-contiguous three-dimensional binding sequence. These substrate positions are therefore not comparable to structures in which the substrate peptide assumes a regular binding conformation. Inhibitor interactions are labelled with an asterisk.

Preference	SDR positions	Evidence
R-3 (55)	17, 82, 86, 127, 158, 162, 185	None, None, Mok et al., 2010, Mok et al., 2010 (CMGC), None, None, None
P+1 (36)	158, 159, 161, 164, 188, 196	Kannan 2004, Zhu et al 2005, Kannan 2004, Zhu et al 2005, Kannan 2004, Kannan 2004
R-2 (27)	27, 162	None, None
P-2 (25)	82, 161, 162, 188, 196	None, Kannan 2004, None, None, Mok et al 2010
L-5 (21)	86, 189	None, Chen et al 2017
R/K+2 (14)	45, 61, 126, 229	None, None, None, None
D/E-2 (13)	157, 189	None, None
D/E-3 (13)	86, 127, 140, 157	None, None, None, None
R-5 (12)	162	None
R/K+3 (10)	161, 237	None, None
D/E+1 (8)	85, 249	None, None
L-2 (8)	131	None
D/E+2 (7)	13, 34	None, None
P+2 (7)	145	None
L+4 (6)	164	None
D/E+4 (6)	73 142 165 249	None, None, None, None

Table S4 - Previous experimental evidence for the predicted SDRs, Related to Figure 2. The SDRs predicted for each preference are listed. For each SDR, the third column gives any available citations for previous literature evidence. The brackets in the first column represent the number of kinases with the given specificity.

REAGENT or RESOURCE	SOURCE	IDENTIFIER
Oligonucleotide to mutate SNF1V244R FW: CCTTTATCGTATGCTTTGTC	This paper	N/A
Oligonucleotide to mutate SNF1V244R RV: ATAACCCCACATGACCACAC	This paper	N/A
Oligonucleotide to mutate SNF1 A218L FW: CAATTATCTTGCTCCTGAAG	This paper	N/A
Oligonucleotide to mutate SNF1 A218L RV: GGAGAACCACAAGAAGTCTT	This paper	N/A
Oligonucleotide to mutate YPK1 Q510G FW: GTGGGACCCCAGGTTACTTGGCACCAGAAC	This paper	N/A
Oligonucleotide to mutate YPK1 Q510G RV: AAAAAGTATCTGTCTTATCATCATCCTTC	This paper	N/A
Oligonucleotide to mutate YPK1 F433H FW: CAATGGTGGTGAGTTGCATTATCATCTACA	This paper	N/A
Oligonucleotide to mutate YPK1 F433 RV: ATAAACGCTAAAACAAAGTATAATTTTTCCG G	This paper	N/A
Oligonucleotide to mutate YPK1 F433K FW: CAATGGTGGTGAGTTGAAATATCATCTACA	This paper	N/A
Oligonucleotide to mutate YPK1 Q537R FW: CTTGTTATACAGAATGCTCACAGGTCTTC	This paper	N/A
Oligonucleotide to mutate YPK1 Q537R RV: ACTCCCAATGTCCACCAATCTACTG	This paper	N/A

Table S6 - Oligonucleotides used to mutate Snf1 and Ypk1, Related to STAR Methods
