# Supplementary material for: Sequence and Structure-Based Analysis of Specificity Determinants in Eukaryotic Protein Kinases
Source: Cell Rep. 2021 Jan 12;34(2):108602. doi: 10.1016/j.celrep.2020.108602 (PMC7809594; doi:10.1016/j.celrep.2020.108602)
Supplement: Document S2. Article plus Supplemental Information [file mmc6.pdf]

Cell Reports

Article

Sequence and Structure-Based Analysis of Specificity Determinants in Eukaryotic Protein Kinases

Graphical Abstract

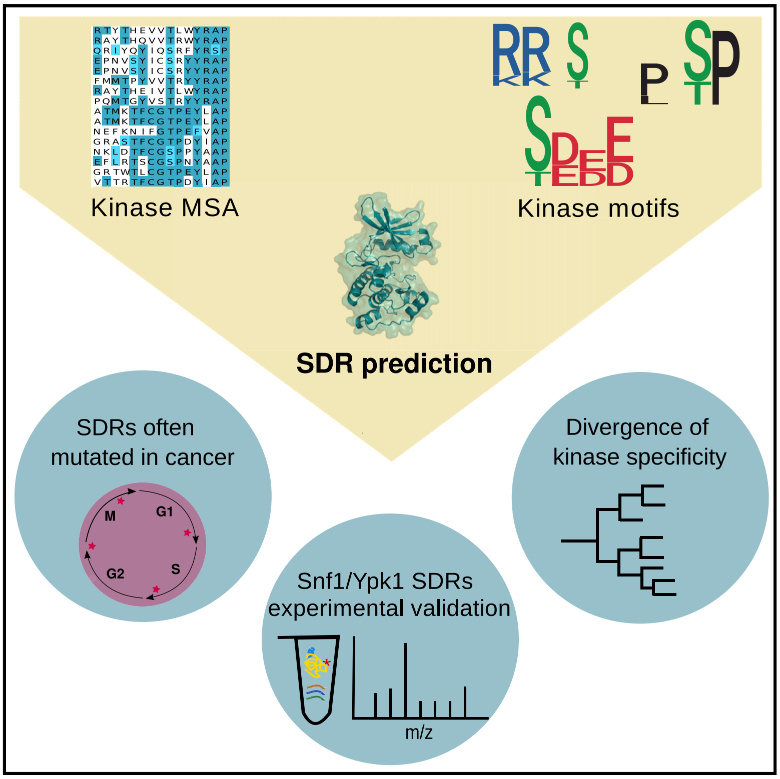

Highlights

- 30 kinase specificity-determining residues (SDRs) predicted across 16 specificities
- SDRs structurally rationalized and experimentally validated
- SDRs often mutated in cancer, with different SDRs targeted in different specificities
- Specificity conserved between orthologs, but SDRs can diverge between paralogs

Authors

David Bradley, Cristina Viéitez, Vinothini Rajeeve, Joel Selkrig, Pedro R. Cutillas, Pedro Beltrao

Correspondence

p.cutillas@qmul.ac.uk (P.R.C.), pbeltrao@ebi.ac.uk (P.B.)

In Brief

Kinase substrate preferences are partly determined by specificity determinants in the kinase domain. However, for many specificities, the determinant residues are unknown. Bradley et al. used a sequence-based approach to predict specificity determinants across many kinases. The residues studied will aid in the interpretation of disease and evolutionary variants.

Bradley et al., 2021, Cell Reports 34, 108602
January 12, 2021 © 2021 The Authors.
https://doi.org/10.1016/j.celrep.2020.108602

CellPress

Cell Reports

CellPress
OPEN ACCESS

Article

Sequence and Structure-Based Analysis of Specificity Determinants in Eukaryotic Protein Kinases

David Bradley,[1,4] Cristina Viéitez,[1,2,4] Vinothini Rajeeve,[3] Joel Selkrig,[2] Pedro R. Cutillas,[3,*] and Pedro Beltrao[1,5,*]
[1]European Molecular Biology Laboratory, European Bioinformatics Institute (EMBL-EBI), Wellcome Genome Campus, Cambridge CB10 1SD, UK
[2]European Molecular Biology Laboratory (EMBL), Genome Biology Unit, 69117 Heidelberg, Germany
[3]Integrative Cell Signalling & Proteomics, Centre for Haemato-Oncology, Barts Cancer Institute, Queen Mary University of London, Charterhouse Square, London EC1M 6BQ, UK
[4]These authors contributed equally
[5]Lead Contact
*Correspondence: p.cutillas@qmul.ac.uk (P.R.C.), pbeltrao@ebi.ac.uk (P.B.)
https://doi.org/10.1016/j.celrep.2020.108602

SUMMARY

Protein kinases lie at the heart of cell-signaling processes and are often mutated in disease. Kinase target recognition at the active site is in part determined by a few amino acids around the phosphoacceptor residue. However, relatively little is known about how most preferences are encoded in the kinase sequence or how these preferences evolved. Here, we used alignment-based approaches to predict 30 specificity-determining residues (SDRs) for 16 preferences. These were studied with structural models and were validated by activity assays of mutant kinases. Cancer mutation data revealed that kinase SDRs are mutated more frequently than catalytic residues. We have observed that, throughout evolution, kinase specificity has been strongly conserved across orthologs but can diverge after gene duplication, as illustrated by the G protein-coupled receptor kinase family. The identified SDRs can be used to predict kinase specificity from sequence and aid in the interpretation of evolutionary or disease-related genomic variants.

INTRODUCTION

Protein post-translational modifications (PTMs) constitute one of the fastest mechanisms of control of protein function, and protein phosphorylation is the most extensive and well-characterized PTM. Protein kinases catalyze the phosphorylation of their target substrates, including other kinases, working in complex signaling networks that are capable of information processing and decision making. These signaling networks are involved in almost all cellular processes, and mutations in protein kinases are often associated with disease (Brognard and Hunter, 2011; Lahiry et al., 2010; Stenberg et al., 2000). In addition, cross-species studies have shown that protein phosphorylation and kinase-substrate interactions can diverge at a very fast pace, suggesting that changes in post-translational control can be a driver of phenotypic diversity (Beltrao et al., 2009; Freschi et al., 2014; Studer et al., 2016). Understanding kinase signaling networks remains a difficult challenge, in particular, because only a small fraction of the known phosphorylation sites can be assigned to their effector kinases.

There are 538 known human protein kinases (Manning et al., 2002), and their specificity of substrate recognition is shaped by the structural and chemical characteristics of both kinase and substrate (Ubersax and Ferrell, 2007). The general fold of different kinases is quite similar, and the specificity of kinases is, in part, determined by changes near the binding pocket. Kinases are thought to recognize a contiguous motif around the phosphosite (4 or 5 amino acids on either side of the P-site) (Amanchy et al., 2007; Knighton et al., 1991; Pearson and Kemp, 1991; Pinna and Ruzzene, 1996) usually called the kinase target motif. These target motif preferences are most often very degenerate, with only a small number of key residues strongly contributing to the recognition.

Knowledge of kinase specificity has been greatly assisted by the development of degenerate peptide libraries that probe the intrinsic specificity of the kinase domain (Songyang et al., 1994, 1996). When applied across many kinases, this technique allows for the identification of domain positions that covary with changes in specificity (Songyang et al., 1996). This approach was used in 2010 to decipher the specificity of 61 yeast kinases and enabled the prediction of several specificity determinants (Mok et al., 2010), and later the identification of a kinase residue defining the phosphoacceptor preference between S and T (Chen et al., 2014). More recently, this method has been applied across kinase family members, such as for kinases in the Nek and STE20 families, to help infer the residues responsible for specificity divergence within families (van de Kooij et al., 2019; Miller et al., 2019). When directed toward kinases belonging

This is an open access article under the CC BY license (http://creativecommons.org/licenses/by/4.0/).

to a functional class, for example, the mitotic kinases, this approach can reveal systems-level mechanisms to ensure signaling fidelity—in this case, by the spatial separation of kinases with overlapping motifs and vice versa (Alexander et al., 2011). The emergence of mass spectrometry (MS) technologies for the systematic profiling of kinase specificity promises to accelerate this field of research even further (Barber et al., 2018; Imamura et al., 2014; Lubner et al., 2018; Sugiyama et al., 2019).

In addition to the intrinsic specificity of the active site, other mechanisms contribute to selectivity, including docking motifs, interaction with protein scaffolds, co-expression, and co-localization (Biondi and Nebreda, 2003; Holland and Cooper, 1999). Sequence analysis has identified 9 kinase groups (AGC, CAMK, CMGC, RGC, TK, TKL, STE, CKI, and “other”), but only a few kinase groups have clear differences in target preferences that are shared with most members of the group. For example, the CMGC kinases tend to phosphorylate serine and threonine residues that have proline at position +1 relative to the phosphoacceptor (Kannan and Neuwald, 2004). However, for most kinase groups, the preferences for residues around the target phosphoacceptor cannot be easily predicted from the primary sequence.

In previous studies of kinase specificity, the analysis of protein structures (Brinkworth et al., 2003; Kobe et al., 2005; Saunders et al., 2008) and machine learning methods (Creixell et al., 2015a) have been used to identify positions within the kinase domain that determine kinase specificity, the specificity-determining residues (SDRs). However, these approaches do not attempt to study the structural basis by which specific target preferences are determined. Methods based on protein kinase alignments can achieve this, but they have only been used to study a few kinase groups so far (Kannan and Neuwald, 2004; Kannan et al., 2007), or they have been restricted to a single model organism (Mok et al., 2010). Here, we have used alignment- and structure-based methods to identify and rationalize the determinants of kinase specificity. We have identified SDRs for 16 target site preferences and show that these can be used to accurately predict kinase specificity. We provide detailed structural characterizations for many determinants and study how these are mutated in cancer or during evolution. We show how the knowledge of SDRs can be combined with ancestral sequence reconstructions to study the evolution of kinase specificity using as an example the G protein-coupled receptor kinase family.

RESULTS

Identification of Kinase Specificity-Determining Residues and Modeling of the Kinase-Substrate Interface

To study kinase target preferences, we compiled a list of 9,084 experimentally validated and unique kinase-phosphosite relations for human, mouse, and yeast kinases. Protein kinase specificities were modeled in the form of position weight matrices (PWMs) for 179 kinases; all phosphosites and PWMs are given in Table S1 and Data S1, respectively. For further analysis, we selected 135 high-confidence PWMs (87 human, 30 mouse, 18 yeast) that could discriminate well between target and non-target phosphorylation sites (see Method Details). For serine/threonine kinases, consistent evidence of active site selectivity is broadly apparent for the −3 and +1 positions relative to the phosphoacceptor, and to a lesser extent the −2 position (Figure 1A). These constraints correspond to the well-established preferences for basic side chains (arginine or lysine) at the −3 and/or −2 position, and in most CMGC kinases for proline at the +1 position. Despite examples of strongly selective tyrosine kinase domains (Shah et al., 2016, 2018), the tyrosine kinases in general show little evidence of strict substrate requirements on par with the proline+1 or arginine-3 signatures, which is perhaps linked to their increased reliance on binding modules such as the SH2 or SH3 domain for specificity (Ubersax and Ferrell, 2007). The tyrosine kinases were excluded from any further analysis as there were too few high-quality PWMs (16) for the reliable detection of their SDRs.

With this information, we then investigated the relationship between protein kinases and substrates at the active site using structural models (Figure 1B) and kinase sequence alignments (Figure 1C). We compiled 12 non-redundant serine/threonine crystal structures of kinases in complex with their substrates in addition to 4 serine/threonine autophosphorylation complexes (Xu et al., 2015) (see full list in Table S2). Kinase-substrate homology models for kinases of interest not represented in this compilation of experimental models were also generated. A structural profile of substrate binding from position −5 to position +4 is given in Figure S1. The kinase positions most frequently in contact (within 4 Å) with the target peptide are highlighted also in Figure 1B. When referring to specific amino acids in the kinase, the single-letter code is used followed by the position of the residue based on the Pfam protein kinase domain model (PF00069). These domain positions have been mapped to the human protein kinase A (PKA) sequence in Table S3.

We developed an alignment-based protocol for the semi-automated detection of putative specificity-determining residues (Figure 1C; Method Details). Briefly, the target preferences described as PWMs were clustered to identify groups of kinases with shared preferences at a position of interest. Putative SDRs are then inferred to be those residues that discriminate kinases with the common substrate preference (e.g., proline at the +1 position or P+1) from other kinases (Figure 1C). Using this approach, we identified 30 predicted SDRs for 16 preferences (Figure 2A) found across the sequence/structure of the kinase domain (Figure 2B). Not surprisingly, SDRs tend to cluster near the binding pocket (Figure 2C), with 33% near the substrate (within 4 Å) compared to ∼12% for any kinase position (Fisher $p < 0.01$), which is in line with previous studies of SDRs (Creixell et al., 2015a; Mok et al., 2010). Such distal kinase residues can still play important roles in determining kinase specificity, although the structural mechanisms are less direct than for residues in contact with the substrate.

To assess the accuracy of these SDRs we tested whether these could be used to predict the specificity of kinases from their sequence alone. For this, we built sequence-based classifiers for the 5 preferences supported by at least 20 positive examples in the study dataset: P+1, P-2, R-2, R-3, and

CellPress
OPEN ACCESS

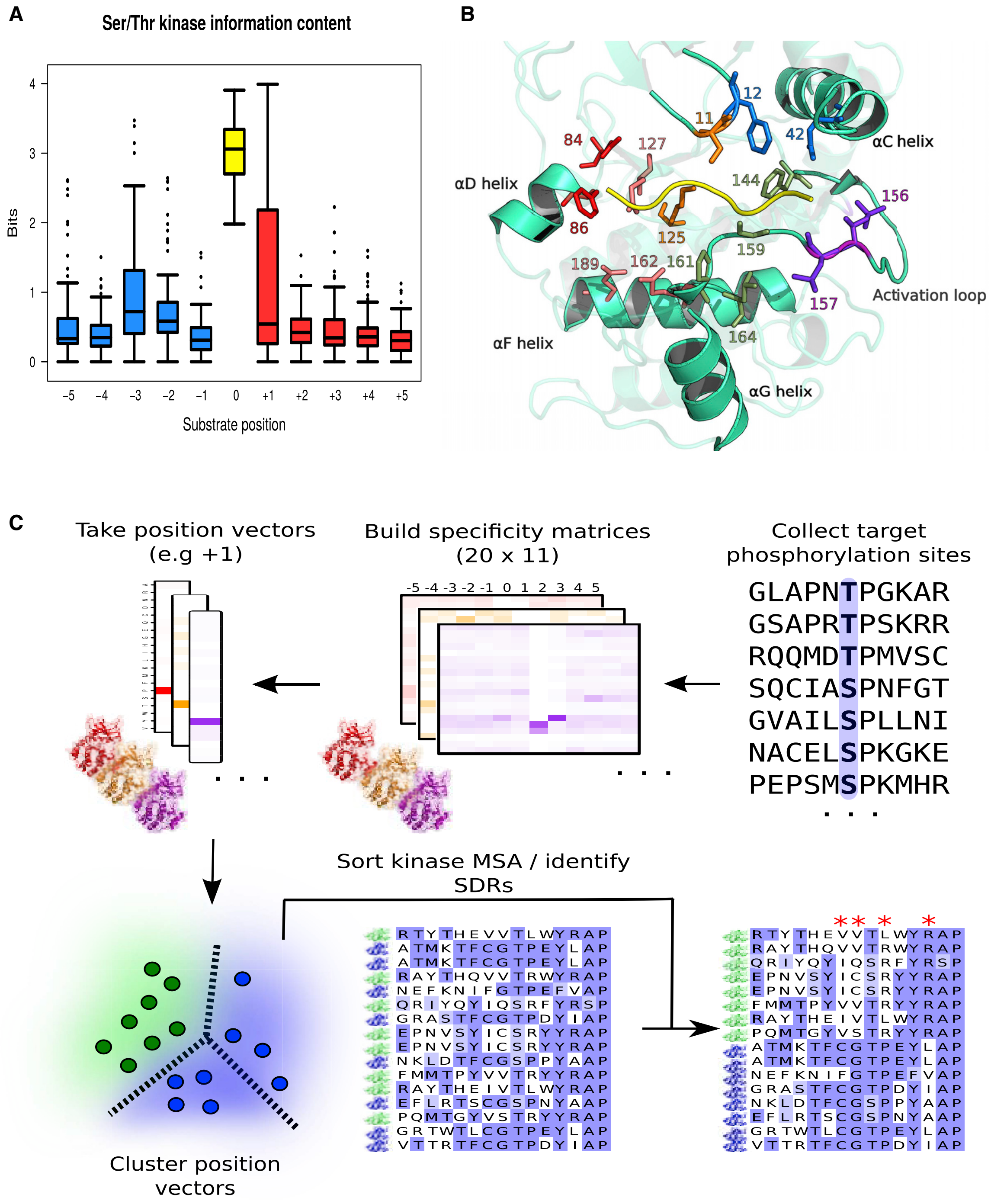

Figure 1. Features of Kinase Target Interaction and Pipeline for SDR Identification

(A) Sequence constraint for substrate positions −5 to +5 for 119 serine/threonine kinases, measured as the bit value for the corresponding column of the kinase PWM.

(B) Interface between a protein kinase (human protein kinase A, PDB: 1ATP) and substrate peptide at the substrate-binding site (Zheng et al., 1993). Kinase residues that commonly bind the substrate peptide (yellow) are represented in stick format and colored according to the corresponding substrate position (−3: red, −2: pink, −1: orange, +1: green, +2: blue, +3: purple). Residue numbering represents the relevant positions of the Pfam protein kinase domain (PF00069).

(C) Semi-automated pipeline for the inference of putative kinase SDRs (specificity-determining residues). The first step involves the construction of many kinase PWMs from known target phosphorylation sites. Vectors corresponding to a substrate position of interest (e.g., +1) are then retrieved from each PWM. An unsupervised learning approach (i.e., clustering) identifies kinases with a common position-based preference (e.g., for proline at +1). Alignment positions that best discriminate kinases belonging to 1 cluster from all others are then identified using computational tools for SDR detection.

A

Preference	SDR positions
R-3 (55)	17, 82, 86, 127, 158, 162, 185
P+1 (36)	158, 159, 161, 164, 188, 196
R-2 (27)	27, 162
P-2 (25)	82, 161, 162, 188, 196
L-5 (21)	86, 189
R/K+2 (14)	45, 61, 126, 229
D/E-2 (13)	157, 189
D/E-3 (13)	86, 127, 140, 157
R-5 (12)	162
R/K+3 (10)	161, 237
D/E+1 (8)	85, 249
L-2 (8)	131
D/E+2 (7)	13, 34
P+2 (7)	145
L+4 (6)	164
D/E+4 (6)	73 142 165 249

B

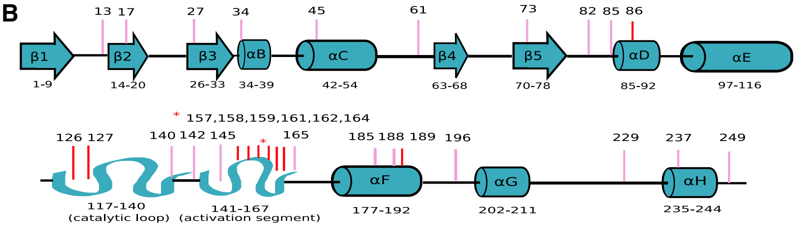

C

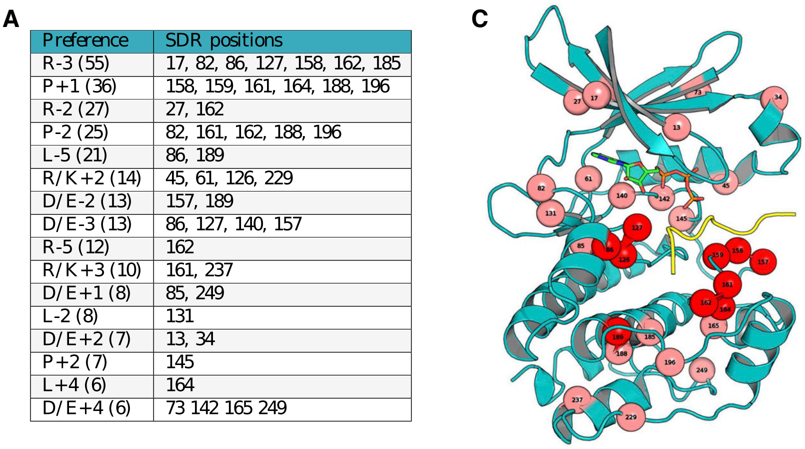

Figure 2. Position of Identified SDRs along the Kinase Sequence and Structure

All putative kinase SDRs from this analysis are (A) listed in a table with their corresponding position preferences, (B) mapped to a 1-dimensional (1D) representation of the kinase secondary structure, and (C) mapped to a kinase-substrate complex structure (PDB: 1ATP). The SDRs colored in dark red (B) and (C) represent positions within 4 Å of the substrate peptide. Residue numbering represents the relevant positions of the Pfam protein kinase domain (PF00069). The numbers in brackets for (A) represent the number of kinases with the given specificity.

L-5. We used a cross-validation procedure in which kinase sequences left out from the model training were later used for testing (see Method Details). These models showed very strong performance with respective cross-validation area under the curve (AUC) values of between 0.83 and 0.99 (Figure S2A). These models also performed well (AUCs between 0.66 and 0.89) when tested against a recent set of experimentally derived PWMs that were not used for training of the models (Sugiyama et al., 2019), showing that the predictions generalize to independent datasets (Figure S2B). Collectively, this shows that for these 5 specificities, the determinant residues can correctly predict the specificity of unseen kinases from their sequence alone, suggesting that the SDRs we have identified are broadly accurate.

Structural Characterization of Kinase SDRs

Most of the predicted SDRs have not been described before and can be further studied by the analysis of structural models. We used available cocrystal structures where possible and also generated homology models, using relevant kinase-substrate structures as a template (see Method Details). Using these models, we could suggest a structural rationale for SDRs of 8 target site preferences that are listed in Figure S3. These include the preferences for arginine at positions −3 and −2; proline at positions −2 and +1; leucine at positions +4 and −5; and aspartate/glutamate at position +1 for AGC and CMGC kinases. Some of the SDRs had been identified in previous studies underscoring the validity of our approach. For example, 4 of the 6 putative SDRs identified here for the proline +1 preference map to the kinase +1 binding pocket (Figure S3) and match determinants described previously (Kannan and Neuwald, 2004). All of the previous literature evidence for the SDRs predicted in Figure 2A is given in Table S4.

We highlight in Figure 3A SDRs for 3 preferences that are less well studied: proline at position −2 (P-2) and leucine at positions +4 (L+4) and −5 (L-5). There are 25 kinases with a modest P-2 preference, including MAPK1, CDK2, and DYRK1A. We identified 5 positions that are putative SDRs for P-2, 2 of which (161 and 162) are proximal (3.4 and 3.7 Å) to the residue in kinase-substrate structures. For position 162, P-2 kinases usually contain a bulky hydrophobic residue (Y or W) that is rarely found in other kinases (Figure S3). Both residues at these positions appear to form hydrophobic contacts with P-2 (Figure 3A). The domain position 161 was also implicated in the preference for the P+1 specificity mentioned above and has been identified as a CMGC-specific determinant (Kannan and Neuwald, 2004). The three other putative determinants—82, 188, and 196—are unlikely to be direct determinants, given their distal position in the protein structure, although we note that 196 was implicated in a previous alignment-based study (Mok et al., 2010). These distal positions may influence the kinase preference through more complex mechanisms such as affecting the dynamics or conformation of the kinase.

We identified 21 kinases (14 CAMK, 5 AGC, 1 CMGC, 1 PRK) with a moderate L-5 preference. Positions 86 and 189 were predicted as determinants in which L-5 kinases are marked by hydrophobic amino acids at position 86 and the absence of glutamate at 189. These residues can be observed to line the hydrophobic −5 position pocket of the MARK2 kinase (Figure 3A). A recent study provided strong evidence for the role of position 189 as an L-5 determinant from a comparative structural analysis of L-5 and R-5 kinases (Chen et al., 2017). This follows from a previous covariation-based approach used to demonstrate that position 144 (DFG+1) helps determine the S versus T phosphoacceptor preference (Chen et al., 2014; Chetty et al., 2020).

For the leucine preference at the +4 position, we identified 6 kinases—MARK2, CAMK1, PRKAA1, PRKAA2 (human), PRKAA1 (mouse), and Snf1 (yeast)—and the domain position 164 as the sole putative SDR. This residue is an alanine in 5 of the kinases listed above (valine in CAMK1). In the MARK2 cocrystal structure, the substrate peptide forms a turn at the +2 position so that the +4

CellPress
OPEN ACCESS

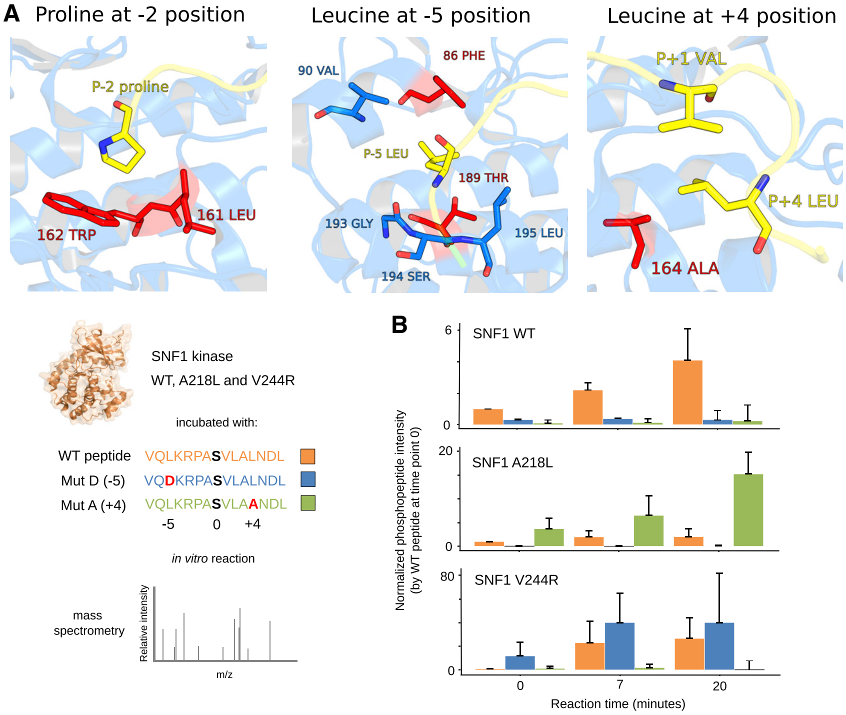

Figure 3. Structural Rationale for Kinase SDRs and Validation Experiments

(A) Kinase-substrate interface for: proline at position −2 (PDB: 2WO6), leucine at position −5 (PDB: 3IEC), and leucine at position +4 (PDB: 3IEC) (Nesić et al., 2010; Soundararajan et al., 2013). The substrate peptides are in yellow and putative SDRs in red. A structural rationalization for each preference is provided briefly in the main text Structural characterization of kinase SDRs, and in Figure S3.

(B) Kinase activity assays for Snf1 wild-type (WT) and 2 mutant versions A218L (the 164 kinase domain position, an L+4 SDR) and V244R (the 189 kinase domain position, an L-5 SDR). The 3 kinases were incubated separately with a known Snf1 target peptide with L at +4 and −5 (orange), as well as the mutant versions A+4 (green) and D-5 (blue). Replicates of *in vitro* reactions were quenched at 0, 7, and 20 min, and the amount of phosphorylation was measured by mass spectrometry. For each kinase and time point, the phosphopeptide intensity relative to the WT peptide at time point zero was calculated, and the median and standard deviation of 3 biological replicates were plotted.

hydrophobic side chain projects toward the kinase pocket of the +1 position and stacks against the +1 residue (Figure 3A). The substitution for alanine in place of residues with aliphatic side chains at position 164 in these kinases therefore seems to generate a small binding pocket that allows the L+4 to functionally substitute for the kinase position 164 by stacking against the +1 residue.

We have selected 2 of the above-described SDRs for experimental characterization (L-5 and L+4). To test these SDRs, we made 2 mutant versions of the Snf1 kinase in yeast: A218L (the 164 kinase position, an L+4 SDR) and V244R (the 189 kinase position, an L-5 SDR) and tested their substrate specificity by *in vitro* kinase assays. Snf1 represents a convenient kinase for this assay as it features both the L-5 and L+4 specificities, although mutation to domain position 86 (F140) was avoided as this would likely affect the R-3 specificity of Snf1 also (Figure S3). Wild-type (WT) Snf1 and these 2 mutants were overexpressed and purified from yeast cells and individually incubated with a Snf1 target peptide of 15 amino acids that contains leucine at +4 and −5 as well as mutant versions with A+4 or D-5. The *in vitro* kinase reactions were quenched at 0, 7, and 20 min, and the amount of peptide phosphorylation was measured by MS (Figures 3B and S4A). As predicted, the A218L Snf1 showed an increased preference for the A+4 peptide but not for the D-5 peptide. The reverse was observed for the V244R Snf1 mutant.

The identification of previously known SDRs, the structural rationale for several of the novel SDRs and the experimental validation of 2 SDRs, further suggests that we have identified positions that are crucial for the recognition of kinases with specific preferences. The SDRs identified here can therefore be used to infer the intrinsic specificity of other kinases belonging to the 5 specificity classes described above (P+1, P-2, R-2, R-3, and L-5) and, as we show below, to study the consequences of mutations within the kinase domain.

Specificity-Determining Residues Are Often Mutated in Cancer

Some kinase SDRs have been observed to be mutated in cancer and congenital diseases (Berthon et al., 2015; Creixell et al., 2015b). Using mutation data from tumor patient samples from The Cancer Genome Atlas (TCGA) (https://cancer.gov/about-nci/organization/ccg/research/structural-genomics/tcga), we have tested for the enrichment of tumor mutations in 4 categories of kinase residues: catalytic, regulatory, SDR (proximal to substrate), and “other” (Figure 4A). SDR residues close (within 4 Å) to the substrate show a significant enrichment of mutations relative to “other” residues in the kinase domain (Mann-Whitney, $p = 6 \times 10^{-4}$; Figure 4B). This enrichment is greater than that observed for catalytic and regulatory sites, highlighting their functional relevance.

We next sought to determine whether the frequency of SDR mutations differs between kinases depending upon their specificity. Given that the specificity models only cover ~20% of all human kinases, we used the SDRs of the 5 most common preferences—P+1, P-2, R-2, R-3, and L-5—to train sequence-based predictors of kinase specificity as described above. Using these models, we annotated all human kinases having a high probability for at least one of these specificities (Table S5). We then compared the frequency of mutations per position for different kinase specificities and found significant differences in the relative mutation frequencies for the P+1 and R-3 positions (represented in Figure 4C). Positions 164 and 161 of the +1 position loop exhibit high levels of differential mutation in the proline-directed kinases.

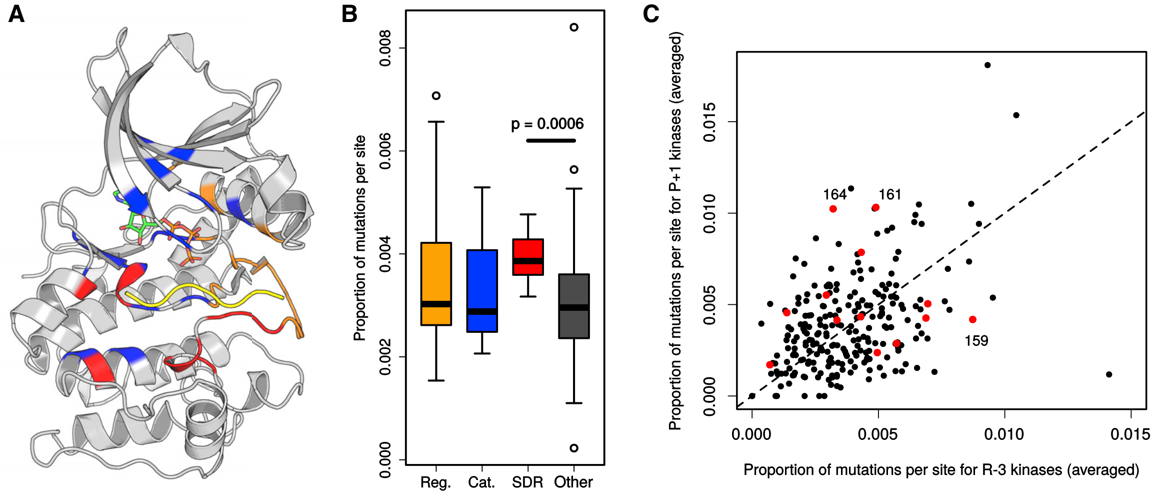

Figure 4. Mutation of SDRs in Cancer

(A) Kinase domain positions are colored according to their functional category (regulatory: orange, catalytic: blue, SDR: red, “other”: gray). The substrate peptide is represented in yellow and ATP in green, orange, and red (PDB: 1ATP).

(B) The fractions of mutations mapping to a given site for a given Ser/Thr kinase were calculated and then averaged across all Ser/Thr kinases. The different sites are grouped according to their functional category. Mutations in SDRs are more frequent than in "other" residues (Mann-Whitney, $p = 6 \times 10^{-4}$).

(C) For a given site, the frequency of mutations in arginine-3 kinases (x axis) and proline+1 kinases (y axis) is plotted. Predicted SDRs are colored in red.

For position 161, the MAP kinases in particular are recurrently mutated in independent samples (MAPK1: 3, MAPK8: 3, MAPK11: 2, MAPK1: 1). This position is known to bind to the phosphotyrosine at 157 that is present in MAPKs (Varjosalo et al., 2013). For the predicted R-3 kinases, the glycine 159 residue of the +1 position pocket is found to be commonly mutated, although this relates not to the R-3 specificity per se but to selectivity against proline at position +1 (Zhu et al., 2005a). Residues 159 and 164 in particular are critical for specificity and highly conserved within the kinase subgroups, such that mutation to any other amino acid would be expected to abrogate P+1 binding. These results suggest that there is a significant recurrence of cancer mutations targeting kinase specificity and not just kinase activity.

The work above illustrates how knowledge of the SDR residues is useful in understanding the functional consequences of cancer mutations. We next studied the changes in SDR residues during the evolution of protein kinases.

Divergence of Kinase Specificity between Orthologs

The full extent to which kinase specificity differs between orthologs is not known (Miller and Turk, 2018; Ochoa et al., 2018). To study this, we compared 65 orthologous pairs between human/mouse kinases and yeast kinases of known specificity. Specificity logos for 2 different examples are given in Figure 5A, indicating that they tend to be similar. We find that the difference in specificity between orthologs (as calculated by the distance between PWMs) is generally similar to that expected for biological replicates of the same kinase ($p = 0.097$, Mann-Whitney, 2-tailed; Figure 5B), but is less than that observed for random human-yeast kinase pairs ($p < 0.01$, Mann-Whitney, 1-tailed; Figure 5B). Only 6/65 (9%) of orthologous pairs (including, e.g., the yeast kinases Cmk1/Cmk2, Sky1, and Pkc1) are more divergent than the median distance of random human-yeast kinase pairs. Kinase specificities are therefore highly conserved in general between human/mouse and *Saccharomyces cerevisiae*, even though they diverged >1 billion years ago (Doolittle et al., 1996).

We next used the identified SDRs to investigate the divergence of specificity between orthologs. We focused our analysis on the 5 specificities we can reliably predict from sequence as described above: P+1, P-2, R-2, R-3, and L-5. Orthologs were retrieved from the Ensembl Genomes Compara database (1,210 species) for each human kinase predicted (Table S5) to have at least 1 of the 5 specificities (i.e., for P+1, P-2, R-2, R-3, or L-5). SDRs for each of the 5 specificities show a much higher sequence conservation than other kinase residues, although lower than was observed for the essential catalytic residues (Figures 5C and S5). Predictions of ortholog specificity, however, suggest that this modest sequence variation among SDRs rarely alters kinase specificity (Figure 5D). Specifically, we predict divergence (posterior probability < 0.5) for only 5% of orthologous groups. In one of the few examples, the Wee2 protein in human features a hydrophobic −5 binding pocket that is present in vertebrate sequences only but not in other species. It is possible that the restricted expression of Wee2 (oocyte-exclusive protein) led to a relaxation of selective constraint on specificity that enabled its evolutionary divergence. For the 5 specificity classes and for *Arabidopsis thaliana* orthologs of human kinases, we predict that the ortholog specificity has diverged in only 12% of cases.

These results demonstrate that kinase active site specificities tend to be highly conserved across orthologs and even between species separated by 1 billion years of evolution.

Divergence of Kinase Specificity within the GRK Family

We then selected the GRK (G protein-coupled receptor kinase) family for a detailed case study of the evolution of target

CellPress
OPEN ACCESS

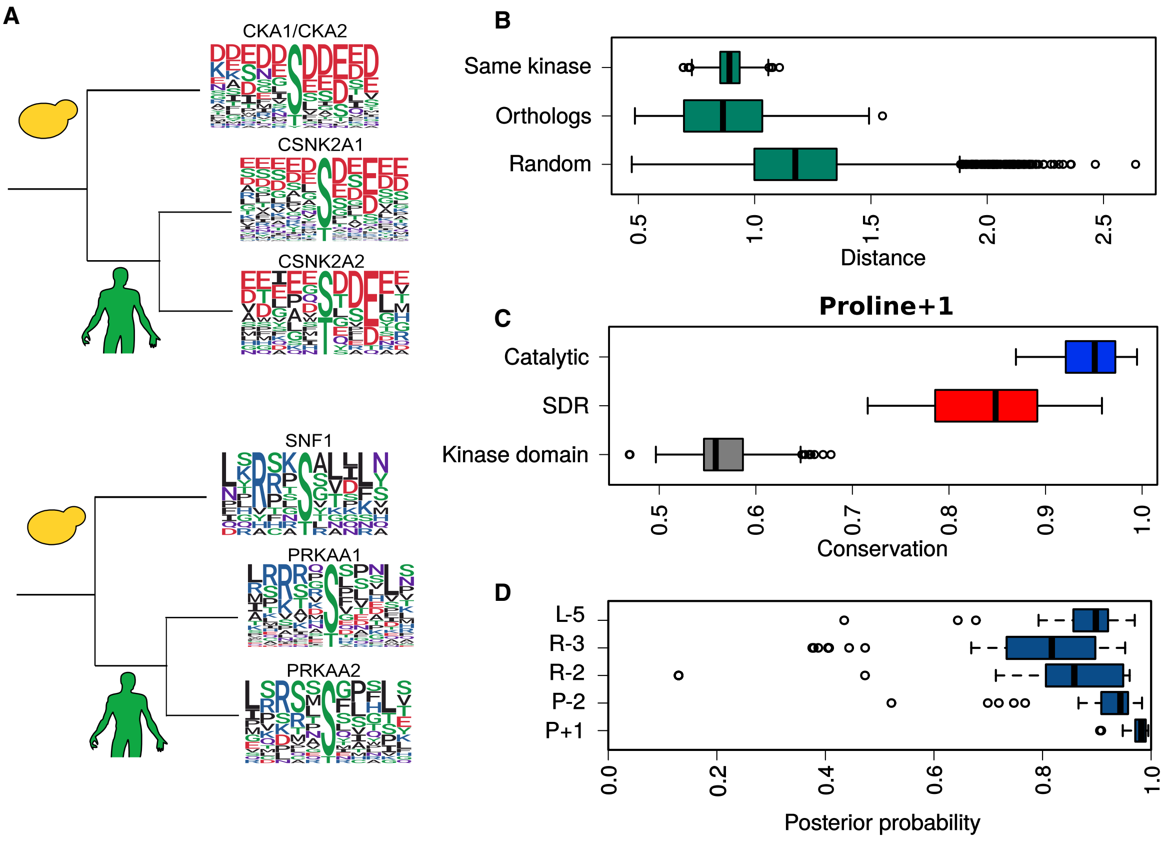

Figure 5. Evolution of Specificity for Orthologous Kinases

(A) Human and yeast kinase specificity logos for 2 different orthologous groups.

(B) Distribution of matrix distances between PWMs generated from phosphosite subsamples of the same kinase (top), orthologous yeast and human/mouse pairs (center), and random human-yeast pairs (bottom).

(C) Conservation of domain residues, SDRs, and catalytic residues for the proline+1 specificity. Each data point represents the average conservation (among kinase domain positions, SDR, or catalytic residues) for an alignment of orthologous kinases in which the human kinase is a predicted proline+1 kinase.

(D) Conservation of specificity for kinases orthologous to human kinases of predicted specificity (L-5, R-3, R-2, P-2, P+1). Each data point represents the average posterior probability (across all kinases in an orthologous group) that the specificity has been conserved.

specificity. The GRK family is 1 of 15 families belonging to the AGC group (Figure 6A) (Manning et al., 2002). However, they have diverged from the characteristic basic residue preferences at positions −2/−5 and −3 of the AGC group (Lodowski et al., 2006). GRK2, for example, is specific for aspartate/glutamate at position −3 (Lodowski et al., 2006; Onorato et al., 1991), and in the GRK5 model presented here, the R-3 signature is absent (Figure 6B). The GRK family is divided into the BARK (β-adrenergic receptor kinase) subfamily, comprising GRK2 (ADRBK1) and GRK3 (ADRBK2) in humans, and the GRK subfamily, comprising GRK1 (rhodopsin kinase), GRK4, GRK5, GRK6, and GRK7 (Manning et al., 2002). We have taken a taxonomically broad sample of 163 GRK kinase sequences to generate a global phylogeny (Figure 6A; Method Details). From this, a maximum-likelihood reconstruction of ancestral sequence states has been performed (Method Details) to study the evolution of substrate preferences on the basis of our detailed understanding of kinase SDRs.

The topology of the tree is in general agreement with a previously published GRK phylogeny (Mushegian et al., 2012). Focusing on the specificity at the −2 and −3 positions (Figures 6C and S6), 2 substitutions between the ancestor of RSK and GRK kinases and the ancestor of all GRK kinases likely caused a reduced preference for arginine at the −3 and −2 positions. The substitution of glutamate forglycine at position 162, an R-3 and R-2 determinant (Figure S3), and the substitution of phenylalanine at position 86 (R-3 determinant), most likely either to histidine or to lysine. From this ancestral node toward the Rhizarian lineage, an additional substitution of glutamate at 189 for arginine likely drove the switch from R-2/R-3 to a novel aspartate/glutamate preference at the −2 position. This 86K/189R pair could be analogous to the 127E/189E pair found in basophilic kinases. In the Heterokont lineages, the histidine/lysine at position 86 in the ancestor of GRK kinases was substituted for serine, and while these kinases retained the 127E/189E pair, the R-2 and R-3 specificities are likely to be attenuated or eliminated, given the substitutions at positions 86 and 162. The BARK kinases had 2 charge-altering substitutions—E127A and E189K—that likely generated the preference for aspartate/glutamate at the −2 and −3 positions, as observed in extant GRK2 kinases

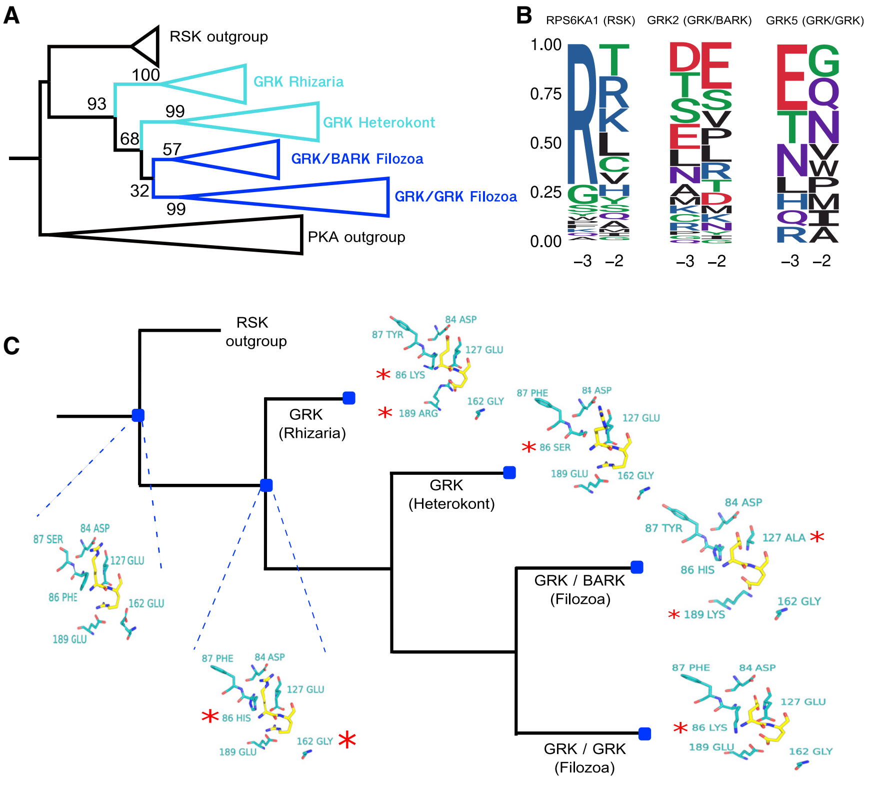

Figure 6. Evolution of GRK Family Specificity

(A) Phylogeny of kinases in the GRK family, including an outgroup of RSK kinases in humans. The supporting number of bootstrap replicates (/100) for relevant clades and bifurcations is represented. The Filozoa represent animals and their closest unicellular relatives, while the Rhizaria and Heterokonts are distantly related protist groups.

(B) Logos at positions −3 and −2 for human RSPS6KA1 (RSK kinase), human GRK2 (GRK/BARK kinase), and human GRK5 (GRK/GRK kinase). Sequence logos were generated from target phosphorylation sites.

(C) Representation of substrate positions −2 and −3 (yellow) and their corresponding kinase binding pockets (cyan) for extant kinases and predicted ancestral sequences. Substitutions in the binding pocket are denoted by a red asterisk.

(Figure 6B). Finally, in the GRK subfamily, a lysine residue (or arginine in GRK1) is usually found at position 86. Notably, no R-2/R-3/R-5 preference is evident for GRK5 (Figure 6B), suggesting that the described substitutions (E162G and F86K) were sufficient to eliminate this specificity.

To experimentally test the divergence of kinase specificity within the GRK family, we selected Ypk1 kinase in yeast as the most similar extant kinase to the RSK-GRK ancestral copy and mutated several amino acids (positions 86, 162, and 189) to mimic evolved versions of the kinase (Figures 6 and S4). We performed kinase assays using synthetic peptides as substrates, as explained above for the Snf1 case. Although the mutations introduced to *YPK1* had an impact on kinase activity, we could observe a decrease in R-5 specificity in the target peptides for one of the mutants (F86H-E162G) mimicking an evolved kinase (Figure S4B; Method Details). This suggests that mutations to positions 86 and 162 together lead to a reduced preference for basic residues at the active site.

The GRK family illustrates how the target preference of a kinase can change after kinase duplication via the substitution of a few key residues. It also illustrates 1 example in which distantly related kinase orthologs may have diverged when comparing the metazoa GRKs to their Rhizaria homologs that diverged ~1.7 billion years ago (Kumar et al., 2017).

DISCUSSION

Here, we have helped to address the challenge of identifying which residues determine kinase preferences toward specific amino acids at specific positions around the target phosphosite. Initial studies of kinase determinants used structures of kinases in complex with target peptides to identify SDRs as being important for substrate binding (Brinkworth et al., 2003; Zhu et al., 2005a). A more recent work has used a machine learning approach to identify SDRs as those that globally maximize the specificity predictive power (Creixell et al., 2015a). These approaches have identified SDR positions but do not assign positions and residues according to specific target preferences (e.g., R-3 or P+1). Alternatively, alignment-based approaches can be used to directly identify residues that contribute to particular preferences but so far have been restricted to 1 kinase group at a time (Kannan and Neuwald, 2004; Kannan et al., 2007) or a single model organism (Mok et al., 2010). We combined a statistical analysis of known kinase targets with alignment- and structure-based approaches to identify and study SDRs. The primary goal of this study was to identify and rationalize SDRs for particular preferences. Importantly, our analysis shows how different positions contribute in unique ways to target site recognition. While we were able to suggest specificity determinants for a large number of previously understudied kinase target preferences, there are still many eukaryotic kinases that do not yet have a known specificity. Kinase specificity is only known for some of the human, mouse, and *S. cerevisiae* kinases. As this knowledge expands, we expect that there will be additional types of kinase specificities beyond those studied here.

It is important to emphasize that some of the predicted SDRs will be likely false positives, given that specificity may correlate with some other kinase property (e.g., kinase regulation, adaptor binding, localization). Here, we demonstrate how a structural analysis can help distinguish likely true positives from false positives. We do not, however, exclude the possibility that distal residues can serve as bona fide determinants, but the structural mechanisms linking distal residues to substrate specificity

remain difficult to study. In addition, some of the structural analysis could yield false negative predictions, given that kinase-substrate cocrystals represent the most stable binding conformations, suggesting that some SDRs may only bind in conformations not observed in the crystal structures.

The *SNF1* mutations of SDRs validated 2 positions contributing to the expected target preferences: position 164 for the L+4 preference and position 189 for the L-5 preference. A recent study also strongly implicated position 189 as an L-5 determinant from a comparative structural analysis (Chen et al., 2017). However, while this residue was mutated and the specificity tested, the mutation of 189 always occurred in combination with other kinase residues, and so the role of position 189 per se as an L-5 SDR was not proven definitively. L+4 specificity, to our knowledge, was thus far uncharacterized and links a traditional +1 determinant (position 164) to a distal substrate position (+4).

The experimental validation in this study was performed with MS on a small number of synthetic peptides *in vitro*. This is a highly sensitive approach that enables the detection of subtle shifts in kinase specificity following mutation. Alternatively, peptide arrays can be used to assay the specificity of mutant kinases (Creixell et al., 2015a; Miller et al., 2019). Peptide arrays can be less sensitive than MS, but they have the advantage of probing for changes in recognition at multiple positions of the target peptide. They have successfully been used to test the effect of mutations on all 20 amino acids at flanking substrate positions and can reveal changes in specificity not predicted *a priori* (Barber et al., 2018; Miller and Turk, 2016).

The study of cancer mutations has revealed that SDRs are commonly mutated as shown by Creixell et al. (2015b). In addition to previous studies, we observed that SDR mutation burden in cancer can reflect kinase specificities, with specific residues being targeted depending on the kinase preference, which we demonstrated here for the P+1 and R-3 specificities. Understanding the impact of mutations in kinases will facilitate the classification of cancer mutations into drivers or passengers, depending on their functional consequences. Our results suggest that grouping all SDR positions, regardless of the kinase specificity, will tend to reduce the accuracy of predicting the impact of mutations, since many SDR positions are only relevant for one or few specificities.

The identification of the SDRs allows us to study the evolution of kinase preferences by ancestral sequence reconstruction. The protein kinase domain has been extensively duplicated throughout evolution, but very little is known about the process of divergence of kinase target preference. We have shown that kinase orthologs tend to maintain their specificity at the active site. This would be expected as they can regulate up to hundreds of targets, and a change in specificity would drastically alter the regulation of a large number of proteins. This high conservation of kinase specificity contrasts with the larger divergence rate of kinase target sites (Beltrao et al., 2009; Freschi et al., 2014; Studer et al., 2016). The evolutionary plasticity of kinase signaling therefore relies primarily on the fast turnover of target sites that can occur without the need for gene duplication.

Examples do still exist, however, of specificity divergence within kinase families. A previous study has shown how the Ime2 kinases (RCK family) have diverged from the other CMGC kinases in their typical preference for proline at the +1 position (Howard et al., 2014). Here, we traced the putative evolutionary history of the GRK family preference at the −2/−3 positions, which demonstrates the divergence of kinase specificity between paralogs and also distantly related orthologs. An understanding of kinase SDRs will allow for further studies of how the variety of target peptide preferences has come about during evolution and the rate at which kinases can switch their preferences after gene duplication.

Kinase target recognition within the cell is complex, and the specificity at the active site is only one of several mechanisms that can determine kinase-substrate interactions (Miller and Turk, 2018; de Oliveira et al., 2016; Ubersax and Ferrell, 2007). Much additional work is needed to establish a global view of kinase target specificity and its evolution.

STAR★METHODS

Detailed methods are provided in the online version of this paper and include the following:

- KEY RESOURCES TABLE
- RESOURCE AVAILABILITY
  - Lead Contact
  - Materials Availability
  - Data and Code Availability
- EXPERIMENTAL MODEL AND SUBJECT DETAILS
- METHOD DETAILS
  - Kinase specificity models
  - Position-based clustering of specificity models
  - Sequence-based prediction of specificity-determining residues (SDRs)
  - Sequence alignment of kinases
  - Identification of kinase-substrate cocrystal structures
  - Structural analysis
  - Kinase-substrate structural models
  - Construction of predictive models and cross-validation
  - Analysis of kinase orthologs
  - Analysis of kinase mutations in cancer
  - GRK phylogeny and ancestral sequence reconstruction
  - Snf1 and Ypk1 mutants construction and *in vitro* kinase assays
  - Mass spectrometry identification of phosphopeptides and quantification
- QUANTIFICATION AND STATISTICAL ANALYSIS

SUPPLEMENTAL INFORMATION

Supplemental Information can be found online at https://doi.org/10.1016/j.celrep.2020.108602.

ACKNOWLEDGMENTS

D.B. received funding from the BBSRC. We would like to thank Omar Wagih for assistance with the kinase-substrate relationship data, Danish Memon for help with TCGA data, and Gerhard Seisenbacher for technical discussion on the

Ypk1 kinase assay. We would also like to thank Romain Studer for useful comments. This work was funded by EMBL core funding.

AUTHOR CONTRIBUTIONS

D.B. and P.B. conceived the study. D.B. and P.B. wrote the manuscript, with contributions from C.V. D.B. performed all of the computational analyses. C.V., D.B., P.B., and P.R.C. planned the experiments. C.V. generated the kinase mutants and performed all of the activity assays, with assistance from J.S. V.R. performed the phosphoproteomics experiments under the supervision of P.R.C.

DECLARATION OF INTERESTS

The authors declare no competing interests.

Received: August 20, 2018
Revised: November 3, 2020
Accepted: December 14, 2020
Published: January 12, 2021

SUPPORTING CITATIONS

The following reference appears in the Supplemental Information: Gibbs and Zoller, 1991; Huang et al., 1995; Moore et al., 2003; Pogacic et al., 2007; Sarno et al., 1997.

REFERENCES

Ahola, V., Aittokallio, T., Vihinen, M., and Uusipaikka, E. (2006). A statistical score for assessing the quality of multiple sequence alignments. BMC Bioinformatics *7*, 484.

Alexander, J., Lim, D., Joughin, B.A., Hegemann, B., Hutchins, J.R.A., Ehrenberger, T., Ivins, F., Sessa, F., Hudecz, O., Nigg, E.A., et al. (2011). Spatial exclusivity combined with positive and negative selection of phosphorylation motifs is the basis for context-dependent mitotic signaling. Sci. Signal. *4*, ra42.

Amanchy, R., Periaswamy, B., Mathivanan, S., Reddy, R., Tattikota, S.G., and Pandey, A. (2007). A curated compendium of phosphorylation motifs. Nat. Biotechnol. *25*, 285–286.

Ashkenazy, H., Penn, O., Doron-Faigenboim, A., Cohen, O., Cannarozzi, G., Zomer, O., and Pupko, T. (2012). FastML: a web server for probabilistic reconstruction of ancestral sequences. Nucleic Acids Res. *40*, W580–W584.

Barber, K.W., Miller, C.J., Jun, J.W., Lou, H.J., Turk, B.E., and Rinehart, J. (2018). Kinase Substrate Profiling Using a Proteome-wide Serine-Oriented Human Peptide Library. Biochemistry *57*, 4717–4725.

Beltrao, P., Trinidad, J.C., Fiedler, D., Roguev, A., Lim, W.A., Shokat, K.M., Burlingame, A.L., and Krogan, N.J. (2009). Evolution of phosphoregulation: comparison of phosphorylation patterns across yeast species. PLOS Biol. *7*, e1000134.

Ben-Shimon, A., and Niv, M.Y. (2011). Deciphering the Arginine-binding preferences at the substrate-binding groove of Ser/Thr kinases by computational surface mapping. PLOS Comput. Biol. *7*, e1002288.

Berthon, A.S., Szarek, E., and Stratakis, C.A. (2015). PRKACA: the catalytic subunit of protein kinase A and adrenocortical tumors. Front. Cell Dev. Biol. *3*, 26.

Biondi, R.M., and Nebreda, A.R. (2003). Signalling specificity of Ser/Thr protein kinases through docking-site-mediated interactions. Biochem. J. *372*, 1–13.

Biondi, R.M., Cheung, P.C.F., Casamayor, A., Deak, M., Currie, R.A., and Alessi, D.R. (2000). Identification of a pocket in the PDK1 kinase domain that interacts with PIF and the C-terminal residues of PKA. EMBO J. *19*, 979–988.

Bodenhofer, U., Kothmeier, A., and Hochreiter, S. (2011). APCluster: an R package for affinity propagation clustering. Bioinformatics *27*, 2463–2464.

Bodenmiller, B., Wanka, S., Kraft, C., Urban, J., Campbell, D., Pedrioli, P.G., Gerrits, B., Picotti, P., Lam, H., Vitek, O., et al. (2010). Phosphoproteomic analysis reveals interconnected system-wide responses to perturbations of kinases and phosphatases in yeast. Sci. Signal. *3*, rs4.

Brinkworth, R.I., Breinl, R.A., and Kobe, B. (2003). Structural basis and prediction of substrate specificity in protein serine/threonine kinases. Proc. Natl. Acad. Sci. USA *100*, 74–79.

Brognard, J., and Hunter, T. (2011). Protein kinase signaling networks in cancer. Curr. Opin. Genet. Dev. *21*, 4–11.

Capella-Gutiérrez, S., Silla-Martínez, J.M., and Gabaldón, T. (2009). trimAl: a tool for automated alignment trimming in large-scale phylogenetic analyses. Bioinformatics *25*, 1972–1973.

Capra, J.A., and Singh, M. (2008). Characterization and prediction of residues determining protein functional specificity. Bioinformatics *24*, 1473–1480.

Chakrabarti, S., and Panchenko, A.R. (2009). Ensemble approach to predict specificity determinants: benchmarking and validation. BMC Bioinformatics *10*, 207.

Chakrabarti, S., Bryant, S.H., and Panchenko, A.R. (2007). Functional specificity lies within the properties and evolutionary changes of amino acids. J. Mol. Biol. *373*, 801–810.

Chakraborty, A., and Chakrabarti, S. (2015). A survey on prediction of specificity-determining sites in proteins. Brief. Bioinform. *16*, 71–88.

Chen, C., Natale, D.A., Finn, R.D., Huang, H., Zhang, J., Wu, C.H., and Mazumder, R. (2011). Representative proteomes: a stable, scalable and unbiased proteome set for sequence analysis and functional annotation. PLOS ONE *6*, e18910.

Chen, C., Ha, B.H., Thévenin, A.F., Lou, H.J., Zhang, R., Yip, K.Y., Peterson, J.R., Gerstein, M., Kim, P.M., Filippakopoulos, P., et al. (2014). Identification of a major determinant for serine-threonine kinase phosphoacceptor specificity. Mol. Cell *53*, 140–147.

Chen, C., Nimlamool, W., Miller, C.J., Lou, H.J., and Turk, B.E. (2017). Rational Redesign of a Functional Protein Kinase-Substrate Interaction. ACS Chem. Biol. *12*, 1194–1198.

Chetty, A.K., Sexton, J.A., Ha, B.H., Turk, B.E., and Boggon, T.J. (2020). Recognition of physiological phosphorylation sites by p21-activated kinase 4. J. Struct. Biol. *211*, 107553.

Creixell, P., Palmeri, A., Miller, C.J., Lou, H.J., Santini, C.C., Nielsen, M., Turk, B.E., and Linding, R. (2015a). Unmasking determinants of specificity in the human kinome. Cell *163*, 187–201.

Creixell, P., Schoof, E.M., Simpson, C.D., Longden, J., Miller, C.J., Lou, H.J., Perryman, L., Cox, T.R., Zivanovic, N., Palmeri, A., et al. (2015b). Kinome-wide decoding of network-attacking mutations rewiring cancer signaling. Cell *163*, 202–217.

de Beer, T.A.P., Berka, K., Thornton, J.M., and Laskowski, R.A. (2014). PDBsum additions. Nucleic Acids Res. *42*, D292–D296.

de Oliveira, P.S.L., Ferraz, F.A.N., Pena, D.A., Pramio, D.T., Morais, F.A., and Schechtman, D. (2016). Revisiting protein kinase-substrate interactions: toward therapeutic development. Sci. Signal. *9*, re3.

Dinkel, H., Chica, C., Via, A., Gould, C.M., Jensen, L.J., Gibson, T.J., and Diella, F. (2011). Phospho.ELM: a database of phosphorylation sites–update 2011. Nucleic Acids Res. *39*, D261–D267.

Doolittle, R.F., Feng, D.F., Tsang, S., Cho, G., and Little, E. (1996). Determining divergence times of the major kingdoms of living organisms with a protein clock. Science *271*, 470–477.

Eddy, S.R. (1998). Profile hidden Markov models. Bioinformatics *14*, 755–763.

El-Gebali, S., Mistry, J., Bateman, A., Eddy, S.R., Luciani, A., Potter, S.C., Qureshi, M., Richardson, L.J., Salazar, G.A., Smart, A., et al. (2019). The Pfam protein families database in 2019. Nucleic Acids Res. *47* (*D1*), D427–D432.

Freschi, L., Osseni, M., and Landry, C.R. (2014). Functional divergence and evolutionary turnover in mammalian phosphoproteomes. PLOS Genet. *10*, e1004062.

Frey, B.J., and Dueck, D. (2007). Clustering by passing messages between data points. Science *315*, 972–976.

Gibbs, C.S., and Zoller, M.J. (1991). Rational scanning mutagenesis of a protein kinase identifies functional regions involved in catalysis and substrate interactions. J. Biol. Chem. *266*, 8923–8931.

Goldberg, J.M., Griggs, A.D., Smith, J.L., Haas, B.J., Wortman, J.R., and Zeng, Q. (2013). Kinannote, a computer program to identify and classify members of the eukaryotic protein kinase superfamily. Bioinformatics *29*, 2387–2394.

Holland, P.M., and Cooper, J.A. (1999). Protein modification: docking sites for kinases. Curr. Biol. *9*, R329–R331.

Holt, L.J., Tuch, B.B., Villén, J., Johnson, A.D., Gygi, S.P., and Morgan, D.O. (2009). Global analysis of Cdk1 substrate phosphorylation sites provides insights into evolution. Science *325*, 1682–1686.

Hornbeck, P.V., Zhang, B., Murray, B., Kornhauser, J.M., Latham, V., and Skrzypek, E. (2015). PhosphoSitePlus, 2014: mutations, PTMs and recalibrations. Nucleic Acids Res. *43*, D512–D520.

Howard, C.J., Hanson-Smith, V., Kennedy, K.J., Miller, C.J., Lou, H.J., Johnson, A.D., Turk, B.E., and Holt, L.J. (2014). Ancestral resurrection reveals evolutionary mechanisms of kinase plasticity. eLife *3*, e04126.

Huang, C.-Y.F., Yuan, C.-J., Blumenthal, D.K., and Graves, D.J. (1995). Identification of the substrate and pseudosubstrate binding sites of phosphorylase kinase γ-subunit. J. Biol. Chem. *270*, 7183–7188.

Imamura, H., Sugiyama, N., Wakabayashi, M., and Ishihama, Y. (2014). Large-scale identification of phosphorylation sites for profiling protein kinase selectivity. J. Proteome Res. *13*, 3410–3419.

Jo, S., Kim, T., Iyer, V.G., and Im, W. (2008). CHARMM-GUI: a web-based graphical user interface for CHARMM. J. Comput. Chem. *29*, 1859–1865.

Kannan, N., and Neuwald, A.F. (2004). Evolutionary constraints associated with functional specificity of the CMGC protein kinases MAPK, CDK, GSK, SRPK, DYRK, and CK2α. Protein Sci. *13*, 2059–2077.

Kannan, N., Haste, N., Taylor, S.S., and Neuwald, A.F. (2007). The hallmark of AGC kinase functional divergence is its C-terminal tail, a cis-acting regulatory module. Proc. Natl. Acad. Sci. USA *104*, 1272–1277.

Katoh, K., Kuma, K., Toh, H., and Miyata, T. (2005). MAFFT version 5: improvement in accuracy of multiple sequence alignment. Nucleic Acids Res. *33*, 511–518.

Kersey, P.J., Allen, J.E., Armean, I., Boddu, S., Bolt, B.J., Carvalho-Silva, D., Christensen, M., Davis, P., Falin, L.J., Grabmueller, C., et al. (2016). Ensembl Genomes 2016: more genomes, more complexity. Nucleic Acids Res. *44* (*D1*), D574–D580.

Knighton, D.R., Zheng, J.H., Ten Eyck, L.F., Ashford, V.A., Xuong, N.H., Taylor, S.S., and Sowadski, J.M. (1991). Crystal structure of the catalytic subunit of cyclic adenosine monophosphate-dependent protein kinase. Science *253*, 407–414.

Kobe, B., Kampmann, T., Forwood, J.K., Listwan, P., and Brinkworth, R.I. (2005). Substrate specificity of protein kinases and computational prediction of substrates. Biochim. Biophys. Acta *1754*, 200–209.

Kumar, S., Stecher, G., Suleski, M., and Hedges, S.B. (2017). TimeTree: A Resource for Timelines, Timetrees, and Divergence Times. Mol. Biol. Evol. *34*, 1812–1819.

Lahiry, P., Torkamani, A., Schork, N.J., and Hegele, R.A. (2010). Kinase mutations in human disease: interpreting genotype-phenotype relationships. Nat. Rev. Genet. *11*, 60–74.

Li, W., and Godzik, A. (2006). Cd-hit: a fast program for clustering and comparing large sets of protein or nucleotide sequences. Bioinformatics *22*, 1658–1659.

Lizcano, J.M., Göransson, O., Toth, R., Deak, M., Morrice, N.A., Boudeau, J., Hawley, S.A., Udd, L., Mäkelä, T.P., Hardie, D.G., and Alessi, D.R. (2004). LKB1 is a master kinase that activates 13 kinases of the AMPK subfamily, including MARK/PAR-1. EMBO J. *23*, 833–843.

Lodowski, D.T., Tesmer, V.M., Benovic, J.L., and Tesmer, J.J.G. (2006). The structure of G protein-coupled receptor kinase (GRK)-6 defines a second lineage of GRKs. J. Biol. Chem. *281*, 16785–16793.

Lubner, J.M., Balsbaugh, J.L., Church, G.M., Chou, M.F., and Schwartz, D. (2018). Characterizing Protein Kinase Substrate Specificity Using the Proteomic Peptide Library (ProPeL) Approach. Curr. Protoc. Chem. Biol. *10*, e38.

Manning, G., Whyte, D.B., Martinez, R., Hunter, T., and Sudarsanam, S. (2002). The protein kinase complement of the human genome. Science *298*, 1912–1934.

Miller, C.J., and Turk, B.E. (2016). Rapid Identification of Protein Kinase Phosphorylation Site Motifs Using Combinatorial Peptide Libraries. Methods Mol. Biol. *1360*, 203–216.

Miller, C.J., and Turk, B.E. (2018). Homing in: Mechanisms of Substrate Targeting by Protein Kinases. Trends Biochem. Sci. *43*, 380–394.

Miller, M.L., Jensen, L.J., Diella, F., Jørgensen, C., Tinti, M., Li, L., Hsiung, M., Parker, S.A., Bordeaux, J., Sicheritz-Ponten, T., et al. (2008). Linear motif atlas for phosphorylation-dependent signaling. Sci. Signal. *1*, ra2.

Miller, C.J., Lou, H.J., Simpson, C., van de Kooij, B., Ha, B.H., Fisher, O.S., Pirman, N.L., Boggon, T.J., Rinehart, J., Yaffe, M.B., et al. (2019). Comprehensive profiling of the STE20 kinase family defines features essential for selective substrate targeting and signaling output. PLOS Biol. *17*, e2006540.

Mir, S., Alhroub, Y., Anyango, S., Armstrong, D.R., Berrisford, J.M., Clark, A.R., Conroy, M.J., Dana, J.M., Deshpande, M., Gupta, D., et al. (2018). PDBe: towards reusable data delivery infrastructure at protein data bank in Europe. Nucleic Acids Res. *46* (*D1*), D486–D492.

Mok, J., Kim, P.M., Lam, H.Y.K., Piccirillo, S., Zhou, X., Jeschke, G.R., Sheridan, D.L., Parker, S.A., Desai, V., Jwa, M., et al. (2010). Deciphering protein kinase specificity through large-scale analysis of yeast phosphorylation site motifs. Sci. Signal. *3*, ra12.

Moore, M.J., Adams, J.A., and Taylor, S.S. (2003). Structural basis for peptide binding in protein kinase A. Role of glutamic acid 203 and tyrosine 204 in the peptide-positioning loop. J. Biol. Chem. *278*, 10613–10618.

Mushegian, A., Gurevich, V.V., and Gurevich, E.V. (2012). The origin and evolution of G protein-coupled receptor kinases. PLOS ONE *7*, e33806.

Nesić, D., Miller, M.C., Quinkert, Z.T., Stein, M., Chait, B.T., and Stebbins, C.E. (2010). Helicobacter pylori CagA inhibits PAR1-MARK family kinases by mimicking host substrates. Nat. Struct. Mol. Biol. *17*, 130–132.

Nuin, P.A.S., Wang, Z., and Tillier, E.R.M. (2006). The accuracy of several multiple sequence alignment programs for proteins. BMC Bioinformatics *7*, 471.

Ochoa, D., Bradley, D., and Beltrao, P. (2018). Evolution, dynamics and dysregulation of kinase signalling. Curr. Opin. Struct. Biol. *48*, 133–140.

Okuno, S., Kitani, T., and Fujisawa, H. (1997). Studies on the substrate specificity of Ca/calmodulin-dependent protein kinase kinase. Neurosci. Res. *28*, S92.

Onorato, J.J., Palczewski, K., Regan, J.W., Caron, M.G., Lefkowitz, R.J., and Benovic, J.L. (1991). Role of acidic amino acids in peptide substrates of the beta-adrenergic receptor kinase and rhodopsin kinase. Biochemistry *30*, 5118–5125.

Pearson, R.B., and Kemp, B.E. (1991). Protein kinase phosphorylation site sequences and consensus specificity motifs: tabulations. Methods Enzymol. *200*, 62–81.

Phillips, J.C., Braun, R., Wang, W., Gumbart, J., Tajkhorshid, E., Villa, E., Chipot, C., Skeel, R.D., Kalé, L., and Schulten, K. (2005). Scalable molecular dynamics with NAMD. J. Comput. Chem. *26*, 1781–1802.

Pike, A.C.W., Rellos, P., Niesen, F.H., Turnbull, A., Oliver, A.W., Parker, S.A., Turk, B.E., Pearl, L.H., and Knapp, S. (2008). Activation segment dimerization: a mechanism for kinase autophosphorylation of non-consensus sites. EMBO J. *27*, 704–714.

Pinna, L.A., and Ruzzene, M. (1996). How do protein kinases recognize their substrates? Biochim. Biophys. Acta *1314*, 191–225.

Pogacic, V., Bullock, A.N., Fedorov, O., Filippakopoulos, P., Gasser, C., Biondi, A., Meyer-Monard, S., Knapp, S., and Schwaller, J. (2007). Structural analysis identifies imidazo[1,2-b]pyridazines as PIM kinase inhibitors with in vitro antileukemic activity. Cancer Res. *67*, 6916–6924.

Prasad, T.S.K., Kandasamy, K., and Pandey, A. (2009). Human Protein Reference Database and Human Proteinpedia as discovery tools for systems biology. Methods Mol. Biol. *577*, 67–79.

Sadowski, I., Breitkreutz, B.-J., Stark, C., Su, T.-C., Dahabieh, M., Raithatha, S., Bernhard, W., Oughtred, R., Dolinski, K., Barreto, K., and Tyers, M. (2013). The PhosphoGRID Saccharomyces cerevisiae protein phosphorylation site database: version 2.0 update. Database (Oxford) *2013*, bat026.

Sarno, S., Vaglio, P., Marin, O., Issinger, O.G., Ruffato, K., and Pinna, L.A. (1997). Mutational analysis of residues implicated in the interaction between protein kinase CK2 and peptide substrates. Biochemistry *36*, 11717–11724.

Saunders, N.F.W., Brinkworth, R.I., Huber, T., Kemp, B.E., and Kobe, B. (2008). Predikin and PredikinDB: a computational framework for the prediction of protein kinase peptide specificity and an associated database of phosphorylation sites. BMC Bioinformatics *9*, 245.

Shah, N.H., Wang, Q., Yan, Q., Karandur, D., Kadlecek, T.A., Fallahee, I.R., Russ, W.P., Ranganathan, R., Weiss, A., and Kuriyan, J. (2016). An electrostatic selection mechanism controls sequential kinase signaling downstream of the T cell receptor. eLife *5*, e20105.

Shah, N.H., Löbel, M., Weiss, A., and Kuriyan, J. (2018). Fine-tuning of substrate preferences of the Src-family kinase Lck revealed through a high-throughput specificity screen. eLife *7*, e35190.

Shaw, R.J., Kosmatka, M., Bardeesy, N., Hurley, R.L., Witters, L.A., DePinho, R.A., and Cantley, L.C. (2004). The tumor suppressor LKB1 kinase directly activates AMP-activated kinase and regulates apoptosis in response to energy stress. Proc. Natl. Acad. Sci. USA *101*, 3329–3335.

Skjærven, L., Jariwala, S., Yao, X.-Q., Idé, J., and Grant, B.J. (2016). The Bio-l.3D Project: Interactive Tools for Structural Bioinformatics. Biophys. J. *110*, 379a.

Songyang, Z., Blechner, S., Hoagland, N., Hoekstra, M.F., Piwnica-Worms, H., and Cantley, L.C. (1994). Use of an oriented peptide library to determine the optimal substrates of protein kinases. Curr. Biol. *4*, 973–982.

Songyang, Z., Lu, K.P., Kwon, Y.T., Tsai, L.H., Filhol, O., Cochet, C., Brickey, D.A., Soderling, T.R., Bartleson, C., Graves, D.J., et al. (1996). A structural basis for substrate specificities of protein Ser/Thr kinases: primary sequence preference of casein kinases I and II, NIMA, phosphorylase kinase, calmodulin-dependent kinase II, CDK5, and Erk1. Mol. Cell. Biol. *16*, 6486–6493.

Soundararajan, M., Roos, A.K., Savitsky, P., Filippakopoulos, P., Kettenbach, A.N., Olsen, J.V., Gerber, S.A., Eswaran, J., Knapp, S., and Elkins, J.M. (2013). Structures of Down syndrome kinases, DYRKs, reveal mechanisms of kinase activation and substrate recognition. Structure *21*, 986–996.

Stamatakis, A. (2014). RAxML version 8: a tool for phylogenetic analysis and post-analysis of large phylogenies. Bioinformatics *30*, 1312–1313.

Stenberg, K.A., Riikonen, P.T., and Vihinen, M. (2000). KinMutBase, a database of human disease-causing protein kinase mutations. Nucleic Acids Res. *28*, 369–371.

Studer, R.A., Rodriguez-Mias, R.A., Haas, K.M., Hsu, J.I., Viéitez, C., Solé, C., Swaney, D.L., Stanford, L.B., Liachko, I., Böttcher, R., et al. (2016). Evolution of protein phosphorylation across 18 fungal species. Science *354*, 229–232.

Sugiyama, N., Imamura, H., and Ishihama, Y. (2019). Large-scale Discovery of Substrates of the Human Kinome. Sci. Rep. *9*, 10503.

Ubersax, J.A., and Ferrell, J.E., Jr. (2007). Mechanisms of specificity in protein phosphorylation. Nat. Rev. Mol. Cell Biol. *8*, 530–541.

van de Kooij, B., Creixell, P., van Vlimmeren, A., Joughin, B.A., Miller, C.J., Haider, N., Simpson, C.D., Linding, R., Stambolic, V., Turk, B.E., and Yaffe, M.B. (2019). Comprehensive substrate specificity profiling of the human Nek kinome reveals unexpected signaling outputs. eLife *8*, e44635.

Varjosalo, M., Keskitalo, S., Van Drogen, A., Nurkkala, H., Vichalkovski, A., Aebersold, R., and Gstaiger, M. (2013). The protein interaction landscape of the human CMGC kinase group. Cell Rep. *3*, 1306–1320.

Velankar, S., Dana, J.M., Jacobsen, J., van Ginkel, G., Gane, P.J., Luo, J., Oldfield, T.J., O'Donovan, C., Martin, M.-J., and Kleywegt, G.J. (2013). SIFTS: Structure Integration with Function, Taxonomy and Sequences resource. Nucleic Acids Res. *41*, D483–D489.

Viéitez, C., Martínez-Cebrián, G., Solé, C., Böttcher, R., Potel, C.M., Savitski, M.M., Onnebo, S., Fabregat, M., Shilatifard, A., Posas, F., and de Nadal, E. (2020). A genetic analysis reveals novel histone residues required for transcriptional reprogramming upon stress. Nucleic Acids Res. *48*, 3455–3475.

Wagih, O., Reimand, J., and Bader, G.D. (2015). MIMP: predicting the impact of mutations on kinase-substrate phosphorylation. Nat. Methods *12*, 531–533.

Wagih, O., Sugiyama, N., Ishihama, Y., and Beltrao, P. (2016). Uncovering Phosphorylation-Based Specificities through Functional Interaction Networks. Mol. Cell. Proteomics *15*, 236–245.

Xu, Q., Malecka, K.L., Fink, L., Jordan, E.J., Duffy, E., Kolander, S., Peterson, J.R., and Dunbrack, R.L., Jr. (2015). Identifying three-dimensional structures of autophosphorylation complexes in crystals of protein kinases. Sci. Signal. *8*, rs13.

Ye, K., Feenstra, K.A., Heringa, J., Ijzerman, A.P., and Marchiori, E. (2008). Multi-RELIEF: a method to recognize specificity determining residues from multiple sequence alignments using a Machine-Learning approach for feature weighting. Bioinformatics *24*, 18–25.

Zheng, J., Trafny, E.A., Knighton, D.R., Xuong, N.H., Taylor, S.S., Ten Eyck, L.F., and Sowadski, J.M. (1993). 2.2 A refined crystal structure of the catalytic subunit of cAMP-dependent protein kinase complexed with MnATP and a peptide inhibitor. Acta Crystallogr. D Biol. Crystallogr. *49*, 362–365.

Zhu, G., Fujii, K., Belkina, N., Liu, Y., James, M., Herrero, J., and Shaw, S. (2005a). Exceptional disfavor for proline at the P + 1 position among AGC and CAMK kinases establishes reciprocal specificity between them and the proline-directed kinases. J. Biol. Chem. *280*, 10743–10748.

Zhu, G., Fujii, K., Liu, Y., Codrea, V., Herrero, J., and Shaw, S. (2005b). A single pair of acidic residues in the kinase major groove mediates strong substrate preference for P-2 or P-5 arginine in the AGC, CAMK, and STE kinase families. J. Biol. Chem. *280*, 36372–36379.

STAR★METHODS

KEY RESOURCES TABLE

REAGENT or RESOURCE	SOURCE	IDENTIFIER
Antibodies		
Peroxidase Anti-peroxidase Soluble Complex antibody produced in rabbit	Sigma	Cat: P1291; RRID: AB_1079562
Chemicals, Peptides, and Recombinant Proteins		
Trifluoroacetic acid UHPLC-MS (Optigrade)	LGC	Cat# SO-9668-B001
Water for LC-MS (Optigrade)	LGC	Cat# SO-9368-B025
Acetonitrile for LC-MS (Optigrade)	LGC	Cat# SO-9340-B025
Formic Acid	Thermo-Fisher Scientific	Cat# F-1850-PB08
WT: VQLKRPASVLALNDL	AQUA peptide from Sigma	Custom synthesis
L-5: VQDKRPASVLALNDL)	AQUA peptide from Sigma	Custom synthesis
L+4: VQLKRPASVLAANDL	AQUA peptide from Sigma	Custom synthesis
WT: GRPRAASFAEK	AQUA peptide from Sigma	Custom synthesis
E-3: GGPEAASFAEK	AQUA peptide from Sigma	Custom synthesis
E-5: GEPGAASFAEK	AQUA peptide from Sigma	Custom synthesis
E-3 E-5: GEPEAASFAEK	AQUA peptide from Sigma	Custom synthesis
Deposited Data		
Kinase co-crystal structures	Mir et al., 2018	https://www.ebi.ac.uk/pdbe/node/1
PKA-peptide complex	Zheng et al., 1993	PDB: 1ATP
DYRK1A-peptide complex	Soundararajan et al., 2013	PDB: 2WO6
MARK2-cagA complex	Nesić et al., 2010	PDB: 3IEC
Kinase-substrate relationships (PSP)	Hornbeck et al., 2015	https://www.phosphosite.org/homeAction
Kinase-substrate relationships (Phospho.ELM)	Dinkel et al., 2011	http://phospho.elm.eu.org/
Kinase-substrate relationships (HPRD)	Prasad et al., 2009	https://hprd.org/
Kinase-substrate relationships (BioGRID)	Sadowski et al., 2013	https://thebiogrid.org/
Kinase domain HMM	El-Gebali et al., 2019	https://pfam.xfam.org/
Kinase substrates for benchmarking	Sugiyama et al., 2019	PMID: 31324866
Peptide library PWMs (yeast)	Mok et al., 2010	PMID: 20159853
Kinase orthologs	Kersey et al., 2016	https://ensemblgenomes.org/
Cancer genome data	The Cancer Genome Atlas (TCGA)	https://www.cancer.gov/about-nci/organization/ccg/research/structural-genomics/tcga
Representative proteomes (rp35)	Chen et al., 2011	https://proteininformationresource.org/rps/
Kinase families	Manning et al., 2002	http://kinase.com/web/current/
Sequence-structure mappings	Velankar et al., 2013	https://www.ebi.ac.uk/pdbe/docs/sifts/quick.html
Experimental Models: Organisms/Strains		
Saccharomyces cerevisiae: SNF1 KO. Yeast strain used to construct SNF1 point mutants. *(BY4741 MATa SNF1 KO)*	From Yeast KO collection. A gift from Lars Steinmetz Lab (EMBL)	This paper
Saccharomyces cerevisiae: SNF1 WT *(BY4741 MATa SNF1 KO + [pGAL-SNF1-URA3 plasmid])*	This paper (PBY362)	This paper (PBY362)
Saccharomyces cerevisiae: SNF1 A218L *(BY4741 MATa SNF1 KO + [pGAL-SNF1^{A218L}-URA3 plasmid])*	This paper (PBY363)	This paper (PBY363)
Saccharomyces cerevisiae: SNF1 V244R *(BY4741 MATa SNF1 KO + [pGAL-SNF1^{V244R}-URA3 plasmid])*	This paper (PBY364)	This paper (PBY364)

(*Continued on next page*)

Continued		
REAGENT or RESOURCE	SOURCE	IDENTIFIER
Saccharomyces cerevisiae: YPK1 WT *(BY4741 MATa YPK1 KO)*	From Yeast KO collection. A gift from Lars Steinmetz Lab (EMBL)	This paper
Saccharomyces cerevisiae: YPK1 F433H-Q510G *(BY4741 MATa + [YPK1*$^{F433H\text{-}Q510G}$ *- TAP-HIS])*	This paper (PBY929)	This paper (PBY929)
Saccharomyces cerevisiae: YPK1 F433K-Q510G-E537R *(BY4741 MATa + [YPK1*$^{F433K\text{-}Q510G\text{-}E537R}$ *- TAP-HIS])*	This paper (PBY1229)	This paper (PBY1229)
Oligonucleotides		
Primers to mutate SNF1, see Table S6	This paper	N/A
Primers to mutate YPK1, see Table S6	This paper	N/A
Software and Algorithms		
CD-HIT	Li and Godzik, 2006	http://weizhongli-lab.org/cd-hit/
APCluster (R package)	Bodenhofer et al., 2011	https://cran.r-project.org/web/packages/apcluster/index.html
GroupSim	Capra and Singh, 2008	https://compbio.cs.princeton.edu/specificity/
SPEER	Chakrabarti et al., 2007	http://www.hpppi.iicb.res.in/ss/index.html
MultiRelief-3D	Ye et al., 2008	https://www.ibi.vu.nl/programs/multirelief/
MAFFT L-INS-i	Katoh et al., 2005	https://mafft.cbrc.jp/alignment/software/
trimAl	Capella-Gutierrez et al., 2009	http://trimal.cgenomics.org/publications
hmmsearch	Eddy, 1998	https://www.ebi.ac.uk/Tools/hmmer/search/hmmsearch
Bio3D (R package)	Skjærven et al., 2016	http://thegrantlab.org/bio3d/
PDBsum	de Beer et al., 2014	https://www.ebi.ac.uk/thornton-srv/databases/pdbsum/
NAMD	(Phillips et al., 2005)	https://www.ks.uiuc.edu/Research/namd/
RAxML	Stamatakis, 2014	https://cme.h-its.org/exelixis/web/software/raxml/
FastML	Ashkenazy et al., 2012	http://fastml.tau.ac.il/overview.php
Xcalibur	Thermo Fisher Scientific	https://www.thermofisher.com/search/results?query=xcalibur%E2%84%A2&navId=12141&persona=Catalog
Custom code	This paper	https://github.com/DBradley27/kinase_SDR
Other		
EASY-Spray source	Thermo Fisher Scientific	ES801
µ-pre-column: PEPMAP100 C18 5µM 0.3X5MM 5/PK	Thermo Fisher Scientific	160454
analytical column: EASY-SPRAY RSLC C18 2µM, 50CM X 75µM	Thermo Fisher Scientific	ES803

RESOURCE AVAILABILITY

Lead Contact

Further information and requests for resources should be directed to and will be fulfilled by the Lead Contact, Pedro Beltrao (pbeltrao@ebi.ac.uk).

Materials Availability

Yeast strains generated during this study are available upon request

Data and Code Availability

The code and data generated during this study are available on GitHub:
(https://github.com/DBradley27/kinase_SDR).

EXPERIMENTAL MODEL AND SUBJECT DETAILS

All yeast strains *(Saccharomyces cerevisiae)* were grown overnight in synthetic defined (SD) media lacking uracil 30°C, diluted in the morning to OD_{600} 0.1 and grown to exponential phase in synthetic defined (SD) media at 30°C . Cells in exponential phase were used for all the experiments.

METHOD DETAILS

Kinase specificity models

Phosphorylation site data were retrieved from the databases HPRD (human), Phospho.ELM (human), PhosphoGRID (*S. cerevisiae*), and PhosphoSitePlus (human and mouse) (Dinkel et al., 2011; Hornbeck et al., 2015; Prasad et al., 2009; Sadowski et al., 2013). Phosphorylation sites without an annotated upstream kinase or literature reference were removed from the dataset. Phosphorylation sites in PhosphoGRID supported exclusively by the (Bodenmiller et al., 2010) or (Holt et al., 2009) studies were excluded from further analysis as these studies provide only indirect evidence for kinase-substrate relations. Target sites that are likely to be homologous were removed with the CD-HIT program using an 85% sequence identity cut-off (Li and Godzik, 2006). We do not include in this analysis protein kinases of the ‘‘Atypical’’ class, which have little to no sequence homology to canonical eukaryotic protein kinases (Manning et al., 2002).

The dataset was further filtered to remove phosphorylation sites mapping to the activation segment of kinase substrates. The justification for this is twofold. First, it has been observed that kinase autophosphorylation sites at the activation segment often conform poorly to kinase consensus motifs derived from peptide library experiments and/or trans-phosphorylation site data (Miller et al., 2008; Pike et al., 2008). Second, from our preliminary analysis we observed a small number of kinases (CAMKK1, PDK1, and LKB1/STK11) with strong substrate motifs corresponding to the *CG[S/T]P* motifs found in non-CMGC kinase activation segments. However, for the kinases CAMK11 and PDK1, experimental evidence suggests that substrate specificity is determined predominantly by allosteric factors, with only a weak reported affinity between the kinase and consensus substrate peptide (Biondi et al., 2000; Okuno et al., 1997). For LKB1/STK11, while the kinase is able to efficiently phosphorylate substrate activation loop sequences *in vitro* (Lizcano et al., 2004), peptide library results fail to recapitulate any residues from the C-terminal *CG[S/T]P* motif, instead implicating leucine at the −2 position as a substrate determinant (Shaw et al., 2004). These results suggest that the strong *CG[S/T]P* consensus motifs observed are more likely to be artifacts of the functional constraints upon this activation segment motif rather than substrate determinants of specificity.

Specificity matrices for each kinase with at least ten phosphorylation sites were then constructed in the form of a position weight matrix (PWM). This threshold has been used in a previous study (Wagih et al., 2015), where it was found that PWMs constructed using fewer substrates tend to be highly variable. In this study, the PWMs constructed are 20 × 11 matrices with the columns representing substrate positions −5 to +5; each value in the matrix represents the relative amino acid frequency at a substrate position. Cross-validation was used to assess kinase model performance. Briefly, a 10-fold cross-validation procedure was implemented to determine the extent to which each kinase model could successfully discriminate between true positive and true negative phosphorylation sites using a matrix-based scoring function, using the protocol described in Wagih et al*.,* 2016. Kinase PWMs with an average AUC (area under curve) value < 0.60 were excluded from further analysis (Wagih et al., 2016).

Too few tyrosine kinase PWMs remained after these filtering steps and were therefore excluded from any further analysis. For all kinase group/family/subfamily classifications, we used the KinBase data resource (Manning et al., 2002).

Position-based clustering of specificity models

Clustering of the PWMs was performed in a position-based manner for each of the five sites N- and C-terminal to the phosphoacceptor (−5, −4, −3, −2, −1; +1, +2, +3, +4, +5) using the affinity propagation (AP) algorithm (Frey and Dueck, 2007). AP is a graph-based clustering method. For the application here, single column vectors (*20 × 1*) from each kinase PWM constitute nodes in the network, and the negative Euclidean distance between vectors represent edges upon initialisation. AP considers all nodes as potential exemplars upon initialisation, and then uses an iterative procedure to automatically identify the optimal number of clusters and cluster exemplar nodes (Frey and Dueck, 2007). We implemented AP in R using the APCluster package with default parameters for the *apcluster()* clustering function (Bodenhofer et al., 2011).

The position-based clusters generated were subject to further refinement before any further analysis. Non-specific clusters, which we define here as any cluster where the summed mean probability of the top two residues is < 0.30, were filtered from the analysis. Clusters with fewer than 6 constituent kinases were also excluded. We also merged clusters with preferences for the same amino acid or for similar residues, as such in-depth analysis of specificity – for example, comparisons between kinases with moderate +1 proline specificity and strong +1 proline specificity, or between arginine preferences and lysine preferences – are beyond the scope of this investigation. For each remaining specificity cluster we retrieved possible ‘‘false negative’’ kinases by incorporating kinases in clusters for which the maximum vector weight is greater than the 40th percentile of the top cluster preference. We suggest such false negative cluster placement to result from noisy weights for non-preferred residues and/or the presence of non-linear phosphorylation sites in the training data. Finally, potential ‘false positive’ cluster members were designated as those kinases where the preferred residue(s) differs from that of the top three average preferred residues of the cluster, and were subsequently removed from the cluster.

Sequence-based prediction of specificity-determining residues (SDRs)

We used three alignment-based methods (GroupSim, Multi-Relief 3D, SPEER) for the prediction of specificity-determining residues (SDRs). The use of more than a single method was motivated by the finding that ensemble approaches that incorporate predictions from three high-performing methods achieve higher precision values than either two-method predictions or the best-performing single-method predictions when benchmarked (Chakrabarti and Panchenko, 2009). While the use of ensemble approaches tends to lower prediction recall (Chakrabarti and Panchenko, 2009), we decided to prioritise precision over recall here given that the predicted SDRs would later be used to inform naive Bayes classifiers of kinase specificity, and that false positive SDRs would lower prediction accuracy.

The three methods employed here represent the three algorithms with the highest single AUC values when benchmarked against a set of 20 protein family alignments with known specificity determinants (Chakraborty and Chakrabarti, 2015). Moreover, all three methods belong to independent categories of SDR predictor (evolutionary, entropy-based, etc), and so make use of non-redundant prediction methodologies (Chakraborty and Chakrabarti, 2015).

The GroupSim, Multi-Relief 3D, and SPEER methods use distinct schemes for position scoring. We therefore follow the precedent of the Chakrabarti and Panchenko (2009) study and identify as putative SDRs those residues among the top 15 ranked sites across all three methods. Standalone versions of GroupSim and SPEER were employed in the pipeline (Capra and Singh, 2008; Chakrabarti et al., 2007). For Multi-Relief 3D, we generated a custom *R* script for the method on the basis of the algorithm description in Ye et al. (2008).

Sequence alignment of kinases

We implemented a semi-automated pipeline for the MSA-based inference of SDRs in an *R* environment. The inputs to the pipeline are the kinase PWMs and an MSA of all kinase protein sequences. The MAFFT L-INS-i method was used to generate MSAs for this analysis (Katoh et al., 2005); this was the highest-performing method in two independent benchmarks of popular alignment tools (Ahola et al., 2006; Nuin et al., 2006). We used the *trim*Al tool to remove MSA positions containing more than 20% ‘gap’ sites (Capella-Gutiérrez et al., 2009).

The pipeline clusters the kinase specificity models in a position-wise manner (discussed above), and then iteratively predicts SDRs for each cluster identified (e. g. +1 proline preference). This is achieved for each cluster by generating a binary partition of the MSA on the basis of cluster membership, and then using the GroupSim, Multi-Relief 3D, and SPEER methods (discussed above) to predict the most likely SDRs from the MSA partition.

Identification of kinase-substrate cocrystal structures

Multiple steps were used to identify all cocrystal structures in the protein data bank (PDB) with a kinase-substrate/inhibitor interface at the active site (Mir et al., 2018).

For the detection of kinase-substrate complexes, we first used the *hmmsearch* command in HMMER (default parameters) to identify all PDB structures containing a eukaryotic protein kinase domain (PFAM: PF00069) sequence (El-Gebali et al., 2019). All PDB files with at least one additional peptide chain were then selected. To distinguish between active site and allosteric binders, we selected all PDB files with at least one residue in contact with either the HRD catalytic aspartate of the kinase domain (P0 binding) or with the position 159 residue of the kinase activation loop (+1 binding). A lenient cut-off of 6 Angstroms was used for this purpose; the retrieved PDB files were then filtered manually. All non-redundant structures retrieved using this procedure are present in Table S2.

All processing was performed in R with use of the Bio3D package (Skjærven et al., 2016). SIFTS XML files were used for residue-level structure-sequence mappings (Velankar et al., 2013).

Structural analysis

For all of the retrieved kinase-substrate structures, an automated approach was used to identify the kinase substrate-binding residues for the substrate positions −5 to +4 (excluding P0). We used the PDBsum tool to identify all substrate-binding residues (de Beer et al., 2014), and to categorise each contact as either hydrogen-bonded, ionic, or non-bonded (i.e., hydrophobic or van der Waals). The substrate residue in closest proximity to the catalytic aspartate of the kinase HRD motif was identified as P0, and the flanking positions were assigned (−2, −1, +1, +2, etc) accordingly. Tyrosine kinases were not included in this analysis, and so the binding profile presented in Figure S1 represents Ser/Thr kinases only. The binding profile does not include kinase domain positions that bind to the substrate infrequently (< 10% of structures).

Kinase-substrate structural models

Kinase-substrate models were constructed using existing X-ray cocrystal structures as templates. Superposition of the kinase of interest (query) with a template cocrystal structure is used to achieve a plausible positioning of the substrate peptide with reference to the query kinase. The template kinase is then removed and the template peptide mutated *in silico* to the sequence of a known phosphorylation site of the query kinase. After resolving steric clashes between kinase and substrate, the resulting complex is then subject to energy minimization (EM), followed by molecular dynamics (MD) equilibration and production runs.

For all models constructed, the template kinase was chosen as the most similar in sequence to the query of the kinases listed in Table S2. Structural superposition was performed in PyMOL. All necessary input files for EM and MD were prepared using the

web-based CHARMM graphical user interface (CHARMM-GUI) with default parameters (Jo et al., 2008). EM and MD runs were executed with the CHARMM36 force field using the NAMD molecular dynamics tool (Phillips et al., 2005). We imposed a harmonic restraint (force constant 90 kcal/mol/Å^2) on the catalytic aspartate of the HRD motif and on the substrate P0 residue to ensure correct positioning of the phosphoacceptor residue.

In each case, the final model used for analysis was generated by finding a representative set of co-ordinates from the protein trajectory. We used the Bio3D package to generate a Principal Components Analysis (PCA) plot of the substrate peptide trajectory co-ordinates (Skjærven et al., 2016). Partition around medoids (PAM) was then used to cluster *n* PCA component scores, where *n* is the lowest number of components that can account for 70% variation. We selected as the kinase-substrate model the set of peptide co-ordinates that served as the medoid to the terminal cluster (i.e., the cluster of co-ordinates corresponding to the trajectory before the end of simulation).

Construction of predictive models and cross-validation

Naive Bayes (NB) algorithms were used to predict the specificity of protein kinases on the basis of the kinase sequence alone. Five separate classifiers were generated, corresponding to the five preferences–P+1, P-2, R-2, R-3, and L-5–supported by at least 20 kinases. We chose this conservative threshold to enable an adequate sample of amino acids per position and therefore to avoid inaccurate predictions.

Each classifier was trained on the 119 Ser/Thr kinase sequences of known specificity. Kinase PWMs where the relative amino acid frequency (e.g., for arginine at position - 3) is 3-fold greater than the background frequency in the proteome were assigned a ‘positive’ label for model training while all other kinases were assigned a ‘negative’ label. In each case, the prior probability of classification was set to 0.5 so that positive or negative classifications would be equally likely *a priori*. We also set a Laplace correction factor of 0.5 during training to account for the absence of particular amino acids in either positive or negative sets of the training data for a given alignment position.

Leave one-out cross-validation (LOOCV) was then used for each classifier to identify the subset of input SDRs that would optimize the performance of the model on the training data with respect to the AUC. Each classifier was initialised with the putative specificity-determining alignment positions described in Figure 2A.

Using a threshold of 3x (i.e., the relative amino acid frequency must be 3-times greater than the background frequency in the proteome), the following AUCs were calculated following cross-validation: 0.91 (P+1), 0.85 (P-2), 0.83 (R-2), 0.93 (R-3), 0.83 (L-5). Using a threshold of 4x, yielded the following AUCs: 0.99 (P+1), 0.88 (P-2), 0.81 (R-2), 0.93 (R-3), 0.89 (L-5); and for 5x: 0.99 (P+1), 0.95 (P-2), 0.86 (R-2), 0.91 (R-3), 0.81 (L-5). Therefore, the cross-validation procedure was robust to the threshold used.

The input SDRs used were as follows, given by their kinase domain positions:

P+1: 159, 188, 196
P-2: 82, 162, 188
R-2: 127, 162, 189
R-3 (non-CMGC): 82, 86, 127, 162
R-3 (CMGC): 86, 127, 189
L-5: 86, 189

For R-2, positions 127 and 189 were not predicted here as SDRs (Figure 2A) as the methods used for SDR detection considers each alignment position independently of other positions. Both positions however are strongly supported as co-operative SDRs in the literature (Ben-Shimon and Niv, 2011; Zhu et al., 2005b), and are included here for specificity prediction given that their prediction was not possible using current methods. This is the only residue pair that we are aware of where this is the case. While this represents a limitation of the current approach, it would not be feasible to automate the detection of correlated SDR associations given the low sample size of kinases with known specificity, as approximately ∼250x125 residue pair associations would need to be calculated for each specificity.

For R-3, separate models were trained for CMGC and non-CMGC kinases as the binding mode in both cases are independent from each other. Using the same set of SDRs across all kinases would therefore not be appropriate (Figure S3). Differences in substrate binding between CMGC and non-CMGC kinases were also observed by the developers of the *Predikin* web server, and are accounted for when making predictions (Saunders et al., 2008).

The same approach described above was used to benchmark the predictions against a set of 141 recently characterized S/T PWMs (Sugiyama et al., 2019) that were not present in the original training set.

Analysis of kinase orthologs

For the orthology analysis of human, mouse, and yeast kinases, we used the 119 PWMs described in the main text in addition to the 61 yeast specificity matrices presented in Mok et al. (2010). Before further analysis, the pT and pY sites were removed from each of the peptide screening models, and then the matrices were normalized so that all columns sum to 1. Human and mouse orthologs (if any) for each yeast kinase were then identified using the Ensembl Rest API for the Ensembl Genomes Compara resource (Kersey et al., 2016). The Frobenius distance was then calculated for every possible pair of human-yeast and mouse-yeast PWMs. This metric

represents the sum of the squared element-wise distances between two matrices, followed by square rooting. Distances for PWMs of the same kinase were generated by subsampling phosphorylation sites (n = 23) from the same kinase and then calculating all possible pairwise Frobenius distances between them. N = 23 corresponds to the median number of phosphorylation sites used to construct the 119 PWMs presented in the main text. When counting the number of divergent yeast-human/mouse orthologous pairs, specificity models from the Mok et al. (2010) study were not considered if the phosphosite-based model of the same kinase was already present.

For the pan-taxonomic analysis of protein kinase orthologs, orthologous sequences were retrieved automatically from the Ensembl Genomes database using the Ensembl Rest API and were aligned using the MAFFT L-INS-i method (Katoh et al., 2005). Orthologs were only retrieved for human kinases with a > 0.9 probability of belonging to at least one of the P+1, P-2, R-2, R-3, or L-5 classes, as determined using the naive Bayes predictors discussed above. Pseudokinases were filtered from the orthologous sets by identifying substitutions at the 30, 123, and 141 domain positions. For each alignment of kinase orthologs, the *bio3d* substitution matrix was used to assess the conservation of every alignment position (Skjærven et al., 2016)). These values were then averaged across the groups 'SDR', 'Catalytic', and 'Kinase Domain' to generate the values presented in Figures 5C and S5. The 'SDR' group represents the predicted SDRs given in Figure 2A. The 'Catalytic' group is the same as what is listed in the section below. 'Kinase domain' represents the complement of the kinase domain against the 'SDR' and 'Catalytic' groups.

For every sequence in an orthologous MSA, posterior probabilities for the corresponding human specificity were also calculated. These values were then averaged across all sequences within an MSA to quantify the extent of specificity divergence among a group of orthologs. A value of 1.0 would indicate the complete conservation of specificity among all orthologs and vice versa. Each data point in Figure 5D therefore represents the average posterior probability (across all sequences in an MSA) of an ortholog having the same specificity as that predicted for the human ortholog ('P+1', 'R-2′', 'R-3′', etc.)

Analysis of kinase mutations in cancer

Mutation data for primary tumor samples was obtained from The Cancer Genome Atlas (TCGA) (https://www.cancer.gov/about-nci/organization/ccg/research/structural-genomics/tcga). Each kinase mutation was assigned to the correct protein isoform and then mapped to the corresponding kinase domain position. The dataset was then filtered to exclude mutations mapping to tyrosine protein kinases.

All kinase domain positions were categorised as 'SDR', 'Catalytic', 'Regulatory', and 'Other'. Catalytic and regulatory sites were inferred from the literature. 'SDR' sites refers to residues that are both potential SDRs (Figure 2A) and often found in close (within 4Å) contact (Figure 2C dark red) with the substrate peptide. 'Other' refers to every other position in the kinase domain.

The set of residues in each class (given by domain position) is as follows:

Catalytic: 8, 10, 13, 15, 28, 30, 48, 85, 123, 125, 128 129, 130, 131, 140, 141, 186, 190
Regulatory: 44, 52, 63, 121, 122, 142, 144, 145, 146, 147, 148, 149, 150, 151, 152, 155, 156, 157, 158, 165, 166, 167
SDR: 86, 126, 127, 157, 158, 159, 161, 162, 164, 189

A comparison of mutation recurrence per site for P+1 and R-3 kinases is represented in Figure 4C. Per site, we used the proportion of mutations mapping to that site for a given kinase, and then took the average of this value across all kinases of the same specificity. This was preferred to the use of raw mutation frequencies, which would bias the analysis toward highly frequent kinase-specific mutations (e.g., BRAF V600E).

GRK phylogeny and ancestral sequence reconstruction

Protein sequences were first retrieved from a taxonomically-broad set of non-redundant proteomes (representative proteomes) (Chen et al., 2011), and then each representative proteome (rp35) was queried with a hidden Markov model (HMM) of the GRK domain (KinBase) using *HMMsearch* (E = 1e-75) (Eddy, 1998). The subfamily classifications of each GRK were then predicted using Kinannote (Goldberg et al., 2013). Sample sequences of the RSK family kinases, which are the most similar in sequence to the GRKs, were also included as an expected outgroup in the phylogeny, as were two kinases of the basophilic PKA family.

The kinase sequences (GRK kinases plus outgroups) were then aligned using the L-INS-i algorithm of MAFFT (Katoh et al., 2005), and filtered to remove pseudokinases and redundant sequences (97% threshold), resulting in 163 sequences to be used for phylogenetic reconstruction. A maximum likelihood phylogeny was generated with RAxML using a gamma model to account for the heterogeneity of rates between sites. The optimum substitution matrix (LG) for reconstruction was also determined with RAxML using a likelihood-based approach (Stamatakis, 2014). FastML was then used for the ML-based ancestral reconstruction of sequences for all nodes in the phylogeny (Ashkenazy et al., 2012). Sequence probabilities were calculated marginally using a gamma rate model and the LG substitution matrix.

Snf1 and Ypk1 mutants construction and *in vitro* kinase assays

The Snf1 and Ypk1 plasmids from the Yeast Gal ORF collection were used as a template for directed mutagenesis to create the following mutants: Snf1 A218L and Snf1 V244R single mutants; Ypk1 F433H E510G double mutant and Ypk1 F433K, E510Q E537R triple mutant. Wild-type and mutant plasmids were transformed into a BY4741 *SNF1 KO* or *YPK1 KO* strains, respectively.

Snf1 and Ypk1 strains were grown to exponential phase in synthetic defined (SD) media lacking uracil, and protein overexpression was induced with 2% galactose for 8h at 30°C. In both cases, cells were collected by centrifugation at 3200rpm for 5min and kept at −80°C. Yeast cell pellets were resuspended in lysis buffer (20mM Tris pH8, 15mM EDTA pH8, 15mM EGTA pH8 and 0.1% Triton X-100) containing a cocktail of protease (cOmplete, from Roche) and phosphatase inhibitors (PhosSTOP, from Sigma). Cells were broken mechanically using glass beads beating at 4°C. Snf1 or Ypk1 protein-immunoprecipitation were performed using rabbit IgG-Protein A agarose beads (Sigma) with rotation for 2h at 4°C. Agarose beads were washed 4 times with lysis buffer. Kinase assays were performed using AQUA synthetic peptides (Sigma) as shown in Figure S4. Briefly, equal amounts of the indicated synthetic peptides were added to each kinase mutant . Snf1 mutants were assayed with peptides WT (VQLKRPASVLALNDL), L-5 (VQDKRPASVLALNDL) and L+4 (VQLKRPASVLAANDL) and Ypk1 mutants with WT (GRPRAASFAEK), E-3 (GGPEAASFAEK), E-5 (GEPGAASFAEK) and E-3 E-5 (GEPEAASFAEK) peptides. ATP mix (ATP 300 μM, 15 mM MgCl2, 0.5 mM EGTA, 15 mM β-glycerol phosphate, 0.2 mM sodium orthovanadate, 0.3 mM DTT) was added to kinase/substrate mix and incubated at 30°C for 0, 2, 7 and 20 minutes. The reactions were quenched by transferring the reaction mixture onto dry ice at the corresponding times. Ypk1 kinase activity assays (Figure S4C) were performed using the incorporation of γ-32P ATP as a readout, as described before (Viéitez et al., 2020).

Mass spectrometry identification of phosphopeptides and quantification

Kinase reaction products were diluted with 0.1% formic acid in LC-MS grade water and 5 μl of solution (containing 10 pmol of the unmodified peptide substrates) were loaded LC-MS/MS system consisting of a nanoflow ultimate 3000 RSL nano instrument coupled on-line to a Q-Exactive Plus mass spectrometer (Thermo Fisher Scientific). Gradient elution was from 3% to 35% buffer B in 15 min at a flow rate 250 nL/min with buffer A being used to balance the mobile phase (buffer A was 0.1% formic acid in LC-MS grade water and B was 0.1% formic acid in LC-MS grade acetonitrile). The mass spectrometer was controlled by Xcalibur software (version 4.0) and operated in the positive ion mode. The spray voltage was 2 kV and the capillary temperature was set to 255°C. The Q-Exactive Plus was operated in data dependent mode with one survey MS scan followed by 15 MS/MS scans. The full scans were acquired in the mass analyzer at 375- 1500 m/z with the resolution of 70 000, and the MS/MS scans were obtained with a resolution of 17 500. For quantification of each phosphopeptide and its respective unmodified form, the extracted ion chromatograms were integrated using the theoretical masses of ions using a mass tolerance of 5 PWM. Values of area-under-the-curve were obtained manually in Qual browser of Xcalibur software (version 4.0).

QUANTIFICATION AND STATISTICAL ANALYSIS

All statistical analysis was performed in the R computing environment and statistical tests performed are described in the Results and Method Details sections.

Cell Reports, Volume *34*

Supplemental Information

Sequence and Structure-Based Analysis of Specificity Determinants in Eukaryotic Protein Kinases

David Bradley, Cristina Viéitez, Vinothini Rajeeve, Joel Selkrig, Pedro R. Cutillas, and Pedro Beltrao

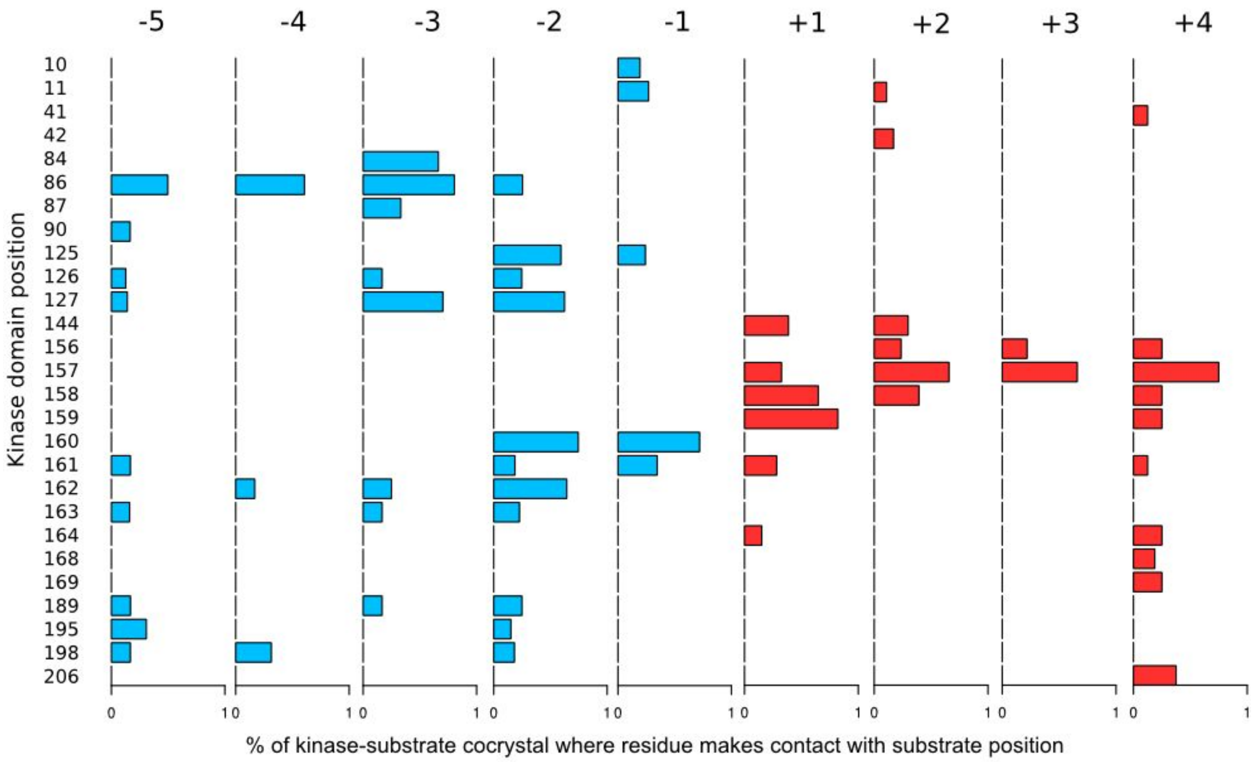

Figure S1. Substrate binding profile of Ser/Thr kinases, Related to Figure 1 - Binding prevalence for Ser/Thr kinase residues (mapped to the eukaryotic protein kinase domain, PFAM: PF00069), in terms of proportion of Ser/Thr kinase-substrate cocrystal structures in which the residue is found to contact (within 4Å) the substrate at a given position (e.g. +1 position). Sets of homologous kinases (e.g. AKT1 and AKT2) were counted once only.

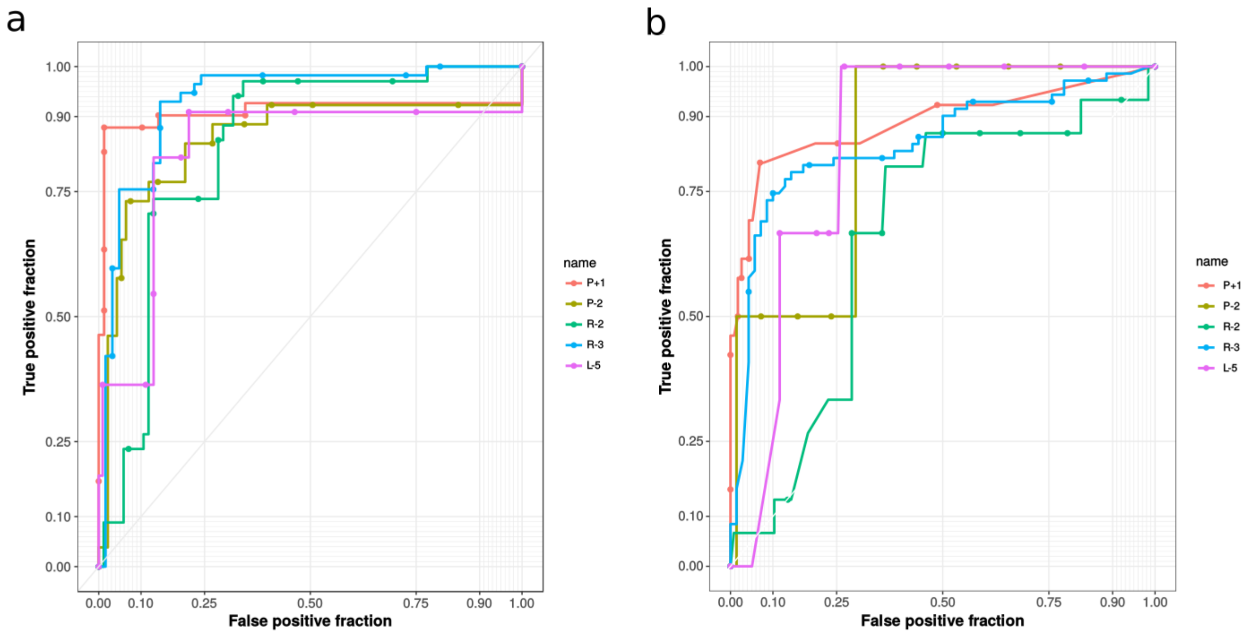

Figure S2. Benchmark performance of naive Bayes specificity predictors, Related to STAR Methods - Receiver Operating Characteristic (ROC) curves for five specificity classifiers. a) Naive Bayes classifiers for P+1, P-2, R-2, R-3, and L-5 preferences were assessed using leave-one-out cross-validation b) Naive Bayes classifiers for P+1, P-2, R-2, R-3, and L-5 preferences were assessed using an independent test set of 141 S/T PWMs from (Sugiyama, Imamura and Ishihama, 2019).

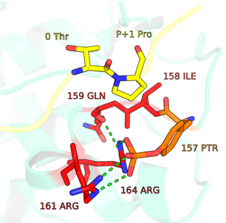	**Proline at position +1:** The +1 proline amide group is unable to act as an H-bond donor to the backbone carbonyl at 159. In absence of this interaction (which occurs in non-Pro+1 kinases with glycine at 159) the arginine side chain at 164 serves as an H-bond donor to the backbone carbonyl at 159. In many Pro+1 kinases the arginine side chain is stabilised by a negative side group (157 pTyr here), which in turn forms polar contacts (2.6Å) with arginine at 161 in this example. Isoleucine at 158 is unlikely to be a direct specificity determinant, but instead probably contributes to the CMGC-specific stabilisation of the kinase via non-bonded contacts with activation segment residues.	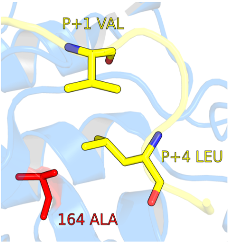	**Leucine at position +4:** Our analysis suggests that a substitution of isoleucine/leucine/methionine for alanine at position 164 contributes to selectivity for leucine at position +4. Mutation to alanine will result in a loss of packing interactions with the hydrophobic side chain at position +1. This is probably compensated for by a hydrophobic residue at position +4, which in the structure PDB: 3IEC can be observed to pack against the +1 valine in the +1 pocket (3.9Å).
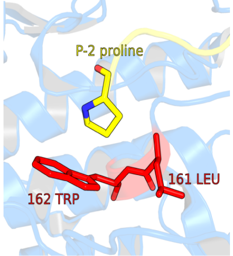	**Proline at position -2:** kinases with this preference usually feature a bulky residue at position 162 (tyrosine or tryptophan), and either leucine or arginine at position 161. Hydrophobic contacts (161: 3.4Å, 162: 3.7Å) between these side chains and the proline side group likely confers modest Pro-2 selectivity. Our analysis also suggests that this preference overlaps with modest Leu-2 selectivity.	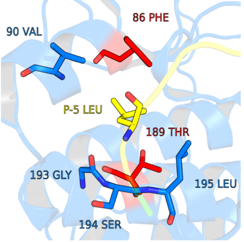	**Leucine at position -5:** The aromatic side chain at position 86 packs against the hydrophobic residue at substrate position -5 (3.4Å). The absence of a negatively-charged glutamate residue at 189 is another factor that favours the binding of a hydrophobic residue at position -5. The other positions highlighted -- 90, 193, 194, 195 -- also constitute the -5 binding pocket and may be important. In addition to the evidence given in **Figure 3**, recent experimental evidence also strongly suggests that position 189 is an SDR for the L-5 specificity (Chen *et al.*, 2017).
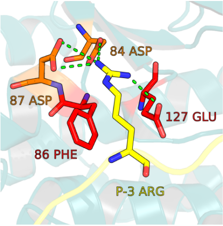	**Arginine at position -3:** position 86 mainly features a tyrosine or phenylalanine residue, which packs against the hydrophobic moiety of the arginine side chain. The glutamate at 127 can be observed to bond with R-3 in a few co-crystal structures in which R-2 or R-5 is not also present. Contact with aspartate/glutamate at 84 (2.8Å) is also observed in many co-crystal structures and has been validated as an SDR (Gibbs and Zoller, 1991; Huang *et al.*, 1995), but is not necessary for R-3 selectivity in all kinases. Polar contacts between R-3 and aspartate at position 87 (2.9Å) have so far been observed in CAMK kinases only (Pogacic *et al.*, 2007; Nesić *et al.*, 2010). In CMGC kinases, the mode of binding is similar to that of R-2 binding in AGC kinases, with glutamates at 127 and 189 (3.0Å and 2.6Å, respectively) forming polar contacts with the substrate arginine.	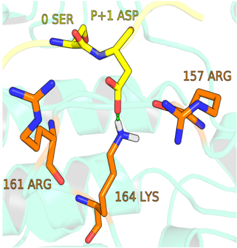	**Aspartate/glutamate at position +1 (CMGC):** The construction of a structural model of a CSNK2A1-peptide complex suggest that positively-charged arginine residues at positions 157 and 161, and a lysine residue at position at 164, are important for aspartate/glutamate selectivity. The suggested role of 164 lysine in particular is supported by a previous experimental study (Sarno *et al.*, 1997).
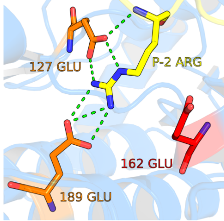	**Arginine at position -2:** Glutamate at positions 127 and 189 are together strongly associated with R-2 selectivity (Zhu *et al.*, 2005; Ben-Shimon and Niv, 2011). The alignment-based method presented here however does not account for positional inter-dependency. Position 162 usually features a glutamate in R-2 kinases, although this residue contacts R-6 directly (2.9Å) rather than R-2. A previous study however suggests a role for 162 in substrate recognition beyond its interaction with R-6 (Moore, Adams and Taylor, 2003).	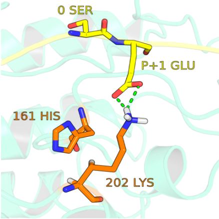	**Aspartate/glutamate at position +1 (AGC):** The construction of a structural model of a GRK2-peptide complex implicates the positively-charged lysine residue at position 202 as a determinant of +1 aspartate/glutamate selectivity. The lysine side chain at position 202 form polar contacts with the glutamate side chain at +1 in the constructed model.

Figure S3. Structural rationalisation of several kinases SDRs, related to Figure 2 and Figure 3 - Eight different kinase position preferences were rationalised using empirical or homology-based (D/E+1 CMGC and D/E+1 AGC) kinase-substrate models. Putative SDRs identified from kinase alignments (**Figure 1c** and **Figure 2**) are coloured in red and all other potential SDRs in orange. Substrate residues are coloured in yellow.

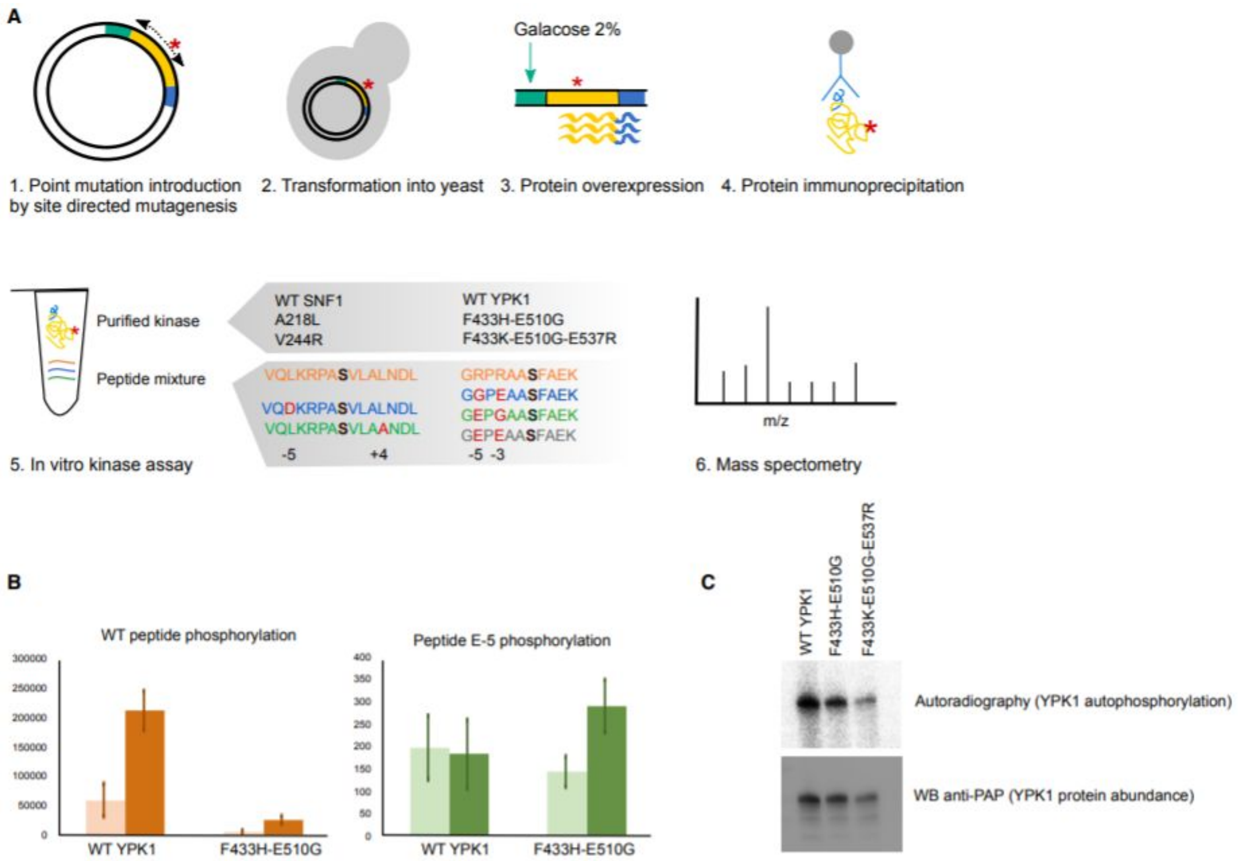

Figure S4. Experimental validation of kinase SDRs in yeast, Related to Figure 3 and Figure 6 - a) Experimental setup used to introduce point mutations in Snf1 and Ypk1-tagged kinases in plasmids, overexpression in yeast, protein purification and in *vitro kinase* assays. For the Ypk1 assay, every kinase (WT or mutant) was individually tested with a pool of peptides: WT (orange), mutated at R-3 (blue), mutated at R-5 (green) and mutated at R-3 and R-5 (grey). Incorporation of γ-32P ATP was measured by mass spectrometry. The residue numbers 433, 510, and 537 correspond to domain positions 86, 162, and 189, respectively. b) Phosphopeptide quantification after the Ypk1 kinase assay for 0 min (light color) and 30 min (dark color) at 30C (median and standard deviation for 3 biological replicates) c) Ypk1 kinase activity shown as kinase autophosphorylation after 30 min incubation with γ-32P ATP (autoradiography). Protein abundance shown by western blot using anti-PAP antibody.

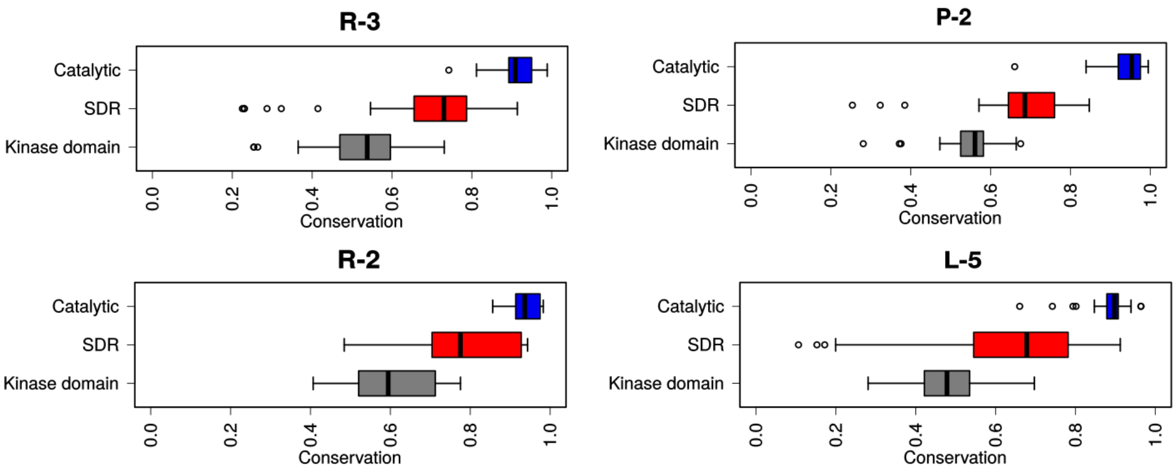

Figure S5. Conservation of kinase SDRs, catalytic residues, and domain residues between orthologs, Related to Figure 5 - Conservation of domain residues, SDRs, and catalytic residues for the R-3, P-2, R-2, and L-5 specificities. Each data point represents the average conservation (among kinase domain positions, SDR, or catalytic residues) for an alignment of orthologous kinases where the human kinase is a predicted R-3, P-2, R-2, or L-5 kinase.

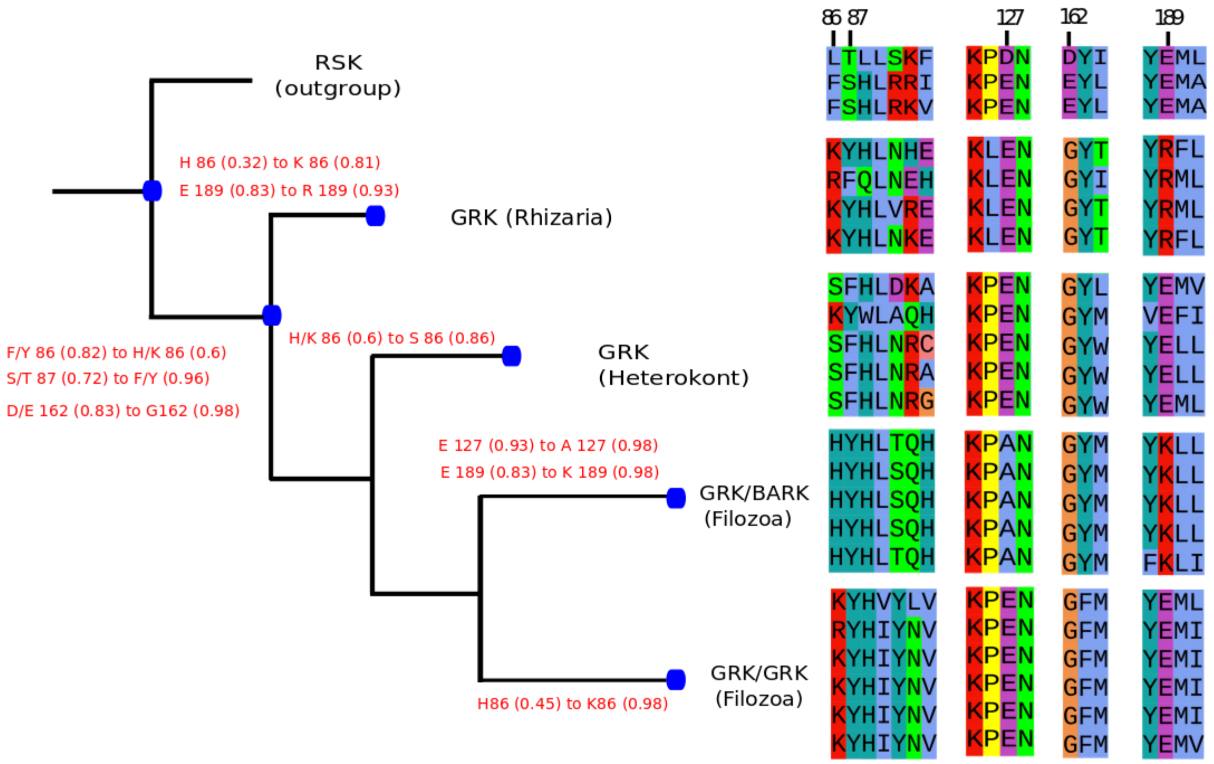

Figure S6. Ancestral sequence reconstruction for the GRK family, related to Figure 6 - Topology of the GRK phylogeny (left) with a sample of the GRK multiple sequence alignment (right). Labels at the selected nodes (blue circles) represent the reconstructed probabilities of amino acids from the antecedent blue node to the labelled node. The labelled domain positions are found in the -2/-3 binding pocket and are discussed in the main text (**Figure 6c**).

PDB	Kinase	Group	Kinase species	Substrate	Resolution (Å)	Publication	-5	-4	-3	-2	-1	0	1	2	3	4	5
2cpk*	PKA	AGC	Human	PKI-alpha	2.7	Knighton *et al.*, 1991	T	G	R	R	N	**A**	I	H	D	x	x
4o21*	PKA	AGC	*M. musculus*	PKI-alpha	1.95	Gerlits *et al.*, 2014	T	G	R	R	A	**S**	I	H	D	x	x
4wih	PKA	AGC	*C. griseus*	PKI-alpha	1.1	Kudlinzki *et al.*, 2015	T	G	R	R	Q	**A**	I	H	D	I	x
4xw5*	PKA	AGC	*M. musculus*	PKI-alpha	1.82	Gerlits *et al.*, 2015	T	G	R	R	A	**C**	I	H	D	x	x
3o7l	PKA	AGC	*M. musculus*	Pln	2.8	Masterson *et al.*, 2010	A	I	R	R	A	**S**	T	I	x	x	x
2phk	PHKg	CAMK	*O. cuniculus*	Synthetic	2.6	Lowe *et al.*, 1997	x	x	R	Q	M	**S**	F	R	L	x	x
1qmz	CDK2	CMGC	Human	Synthetic	2.2	Brown *et al.*, 1999	x	x	H	H	A	**S**	P	R	K	x	x
3qhr	CDK2	CMGC	Human	Synthetic	2.2	Bao *et al.*, 2011	x	x	x	P	K	**T**	P	K	K	A	K
1o6k	AKT-2	AGC	Human	GSK3-beta	1.7	Yang *et al.*, 2002	R	P	R	T	T	**S**	F	A	E	x	x
3cqw	AKT-1	AGC	Human	Synthetic	2.0	Lippa *et al.*, 2008	R	P	R	T	T	**S**	F	A	E	x	x
3mvh	v-AKT	AGC	Human	Synthetic	2.01	Freeman-Cook *et al.*, 2010	R	P	R	T	T	**S**	F	A	E	x	x
2c3i	PIM-1	CAMK	Human	Synthetic	1.9	Pogacic *et al.*, 2007	R	R	R	H	P	**S**	G	x	x	x	x
4dc2	PKC-i	AGC	*M. musculus*	Par-3	2.4	Wang *et al.*, 2012	G	F	G	R	Q	**S**	M	S	x	x	x
2wo6	DYRK-1A	CMGC	Human	Crb-2	2.5	Soundararajan *et al.*, 2013	x	A	R	P	G	**T**	P	A	L	x	x
4jdh	PAK-4	STE	Human	Synthetic	2	Chen *et al.*, 2014	x	x	R	R	R	**T**	W	Y	F	G	G
4l67*	PAK-4	STE	Human	Pak4	2.8	Wang *et al.*, 2013	A	R	R	P	K	**P**	L	V	D	P	A
4ouc	Haspin	Other	Human	Histone H3.2	1.9	Maiolica *et al.*, 2014	x	x	x	A	R	**T**	K	Q	T	A	x
3kl8*	CAMKII	CAMK	*C .elegans*	CAMK2n1	3.37	Chao *et al.*, 2010	I	G	R	S	K	**R**	V	V	I	x	x
3iec*	MARK2	CAMK	*H. sapiens*	cagA	2.2	Nesic *et al.*, 2010	L	K	R	H	D	**K**	V	D	D	L	S
3tl8	BAK1	N/A	*A. thaliana*	HoAB2	2.5	Cheng *et al.*, 2011	I	D	L	G	E	**S**	L	V	Q	H	P

Table S2 - List of PDB structures featuring Ser/Thr protein kinases in complex with a substrate peptide/protein at the active site, Related to STAR Methods. In *4dc2* (PKC-i), the substrate peptide N-terminal to the phosphoacceptor ‘loops out’ from the active site to form a non-contiguous three-dimensional binding sequence. These substrate positions are therefore not comparable to structures in which the substrate peptide assumes a regular binding conformation. Inhibitor interactions are labelled with an asterisk.

Preference	SDR positions	Evidence
R-3 (55)	17, 82, 86, 127, 158, 162, 185	None, None, Mok et al., 2010, Mok et al., 2010 (CMGC), None, None, None
P+1 (36)	158, 159, 161, 164, 188, 196	Kannan 2004, Zhu et al 2005, Kannan 2004, Zhu et al 2005, Kannan 2004, Kannan 2004
R-2 (27)	27, 162	None, None
P-2 (25)	82, 161, 162, 188, 196	None, Kannan 2004, None, None, Mok et al 2010
L-5 (21)	86, 189	None, Chen et al 2017
R/K+2 (14)	45, 61, 126, 229	None, None, None, None
D/E-2 (13)	157, 189	None, None
D/E-3 (13)	86, 127, 140, 157	None, None, None, None
R-5 (12)	162	None
R/K+3 (10)	161, 237	None, None
D/E+1 (8)	85, 249	None, None
L-2 (8)	131	None
D/E+2 (7)	13, 34	None, None
P+2 (7)	145	None
L+4 (6)	164	None
D/E+4 (6)	73 142 165 249	None, None, None, None

Table S4 - Previous experimental evidence for the predicted SDRs, Related to Figure 2. The SDRs predicted for each preference are listed. For each SDR, the third column gives any available citations for previous literature evidence. The brackets in the first column represent the number of kinases with the given specificity.

REAGENT or RESOURCE	SOURCE	IDENTIFIER
Oligonucleotide to mutate SNF1V244R FW: CCTTTATCGTATGCTTTGTC	This paper	N/A
Oligonucleotide to mutate SNF1V244R RV: ATAACCCCACATGACCACAC	This paper	N/A
Oligonucleotide to mutate SNF1 A218L FW: CAATTATCTTGCTCCTGAAG	This paper	N/A
Oligonucleotide to mutate SNF1 A218L RV: GGAGAACCACAAGAAGTCTT	This paper	N/A
Oligonucleotide to mutate YPK1 Q510G FW: GTGGGACCCCAGGTTACTTGGCACCAGAAC	This paper	N/A
Oligonucleotide to mutate YPK1 Q510G RV: AAAAAGTATCTGTCTTATCATCATCCTTC	This paper	N/A
Oligonucleotide to mutate YPK1 F433H FW: CAATGGTGGTGAGTTGCATTATCATCTACA	This paper	N/A
Oligonucleotide to mutate YPK1 F433 RV: ATAAACGCTAAAACAAAGTATAATTTTTCCG G	This paper	N/A
Oligonucleotide to mutate YPK1 F433K FW: CAATGGTGGTGAGTTGAAATATCATCTACA	This paper	N/A
Oligonucleotide to mutate YPK1 Q537R FW: CTTGTTATACAGAATGCTCACAGGTCTTC	This paper	N/A
Oligonucleotide to mutate YPK1 Q537R RV: ACTCCCAATGTCCACCAATCTACTG	This paper	N/A

Table S6 - Oligonucleotides used to mutate Snf1 and Ypk1, Related to STAR Methods
